# Genome-wide Identification, Classification, Expression and Duplication Analysis of *GRAS* Family Genes in *Juglans regia* L.

**DOI:** 10.1038/s41598-019-48287-x

**Published:** 2019-08-12

**Authors:** Shaowen Quan, Jianxin Niu, Li Zhou, Hang Xu, Li Ma, Yang Qin

**Affiliations:** 1 Department of Horticulture, College of Agriculture, Shihezi University, Shihezi, 832003 Xinjiang China; 2 Xinjiang Production and Construction Corps Key Laboratory of Special Fruits and Vegetables Cultivation Physiology and Germplasm Resources Utilization, Shihezi, 832003 Xinjiang China

**Keywords:** Plant molecular biology, Shoot apical meristem

## Abstract

Fifty-two *GRAS* genes are identified in walnut genome. Based on the evolutionary relationship and motif analysis, the walnut *GRAS* gene family was divided into eight subfamilies, and the sequence features analysis of *Jr*GRAS proteins showed that the *Jr*GRAS protein sequences were both conserved and altered during the evolutionary process. Gene duplication analysis indicated that seven *GRAS* genes in walnut have orthologous genes in other species, and five of them occurred duplicated events in walnut genome. Expression pattern analysis of the *GRAS* family genes in walnut showed that two *Jr*GRAS genes (*JrCIGRa-b* and *JrSCL28a*) were differentially expressed between flower bud and leaf bud (p < 0.01), and two *JrGRAS* genes (*JrCIGRa*-*b* and *JrSCL1*3*b*-*d*) were differentially expressed between the different development stages of flower buds transition (p < 0.01), besides, three hub genes (*JrGAIa*, *JrSCL3f* and *JrSHRc*) were identified by co-expression analysis, which suggested these *GRAS* genes may play an important role in regulating the development of apical meristem in walnut. This study laid a foundation for further understanding of the function of *GRAS* family genes in walnut.

## Introduction

*GRAS* genes, derived from the first three members to be identified as a plant-specific gene family, the *GIBBERELLIN-INSENSITIVE* (*GAI*), *Repressor of ga1*-*3* (*RGA*) and *SCARECROW* (*SCR*)^1^. Among them, GAI proteins and RGA proteins are members of the DELLA proteins, which play important roles in repressing gibberellin responses^2^ and jasmonate (JA) and light signaling regulation^3^, and SCR proteins act as a key regulator of Arabidopsis roots^4–6^.

GRAS proteins share conserved domains in their C-terminus, comprised LHR I, VHIID, LHR II, PFYRE and SAW^1,7–9^, however, the N-terminus of GRAS proteins show a great divergence, which may result to the functional specificity of each protein^10^. Although metazoan STATs share similar domain organization with plant GRAS, it is lack of enough support for the hypothesis that GRAS proteins are plant STATS^11^. Recent structural studies have illustrated that the conserved GRAS domain comprises an α-helical cap and α/β core subdomains, which mediates protein-protein interactions^4^.

Up to now, more than a dozen of *GRAS* gene family have been identified, including *Arabidopsis thaliana*^1,7,12^, *Rice*^7,13^, *Populus*^14^, *pine*^15^, *Chinese cabbage*^16^, *tobacco*^17^, *tomato*^18,19^, *Prunus mume*^20^, *Jatropha curcas* L.^21^, *Lotus japonicus*^22^, *grapevine*^23,24^, *Nelumbo nucifera*^25^, *Ricinus communis*^26^, *Betula kirghisorum*^27^, *Isatis indigotica*^28^, *apple*^29^, *Zea mays* L.^30^, *Medicago truncatula*^31^, *Camellia sinensis*^32^ and *Gossypium hirsutum*^33^. The plant-specific GRAS family of proteins function as transcriptional regulators and play critical roles in development and signaling, such as in signal transduction (gibberellin signal transduction^2,34^, phytochrome A signal transduction)^35,36^, stress responses^23,37–40^, meristem formation and maintenance^8,41–44^ and promoting flowering^9^.

Walnut is cultivated worldwide for its nutritious fruits and commercially valuable timber, however, it needs many years before flowering and to become productive^45–47^. Previous research has shown that some of the *GRAS* members play important roles in meristem development^8,41–44^. To better understanding the molecular mechanism of walnut flower bud transition, it is necessary to investigate the *GRAS* family in walnut. With the availability of walnut genome sequences^48^ and transcriptome data of the walnut female flower buds and leaf buds, it is possible for us to identify all the *GRAS* family genes in walnut.

In this study, *GRAS* family genes in walnut have been identified in genome-wide. The phylogenetic relationship, sequence alignment, conserved motif composition and gene duplication of the *JrGRAS* genes were systematically analyzed, and their expression patterns in different tissues (flower bud and leaf bud) and different development stages (before, during, after the flower transition period) were explored using transcriptome data and validated by qRT-PCR experiments. Finally, protein-protein interactions analysis was conducted to investigate how they participate in diverse functions by interacting with other proteins. This research lay a foundation for further function investigations of *GRAS* genes in walnut.

## Results

### Identification of *GRAS* family members in walnut

A total of seventy protein sequences (include protein isoforms) encoded by fifty-two genes, which including the GRAS domain were identified as the walnut GRAS proteins for further analysis. Fifty-two *GRAS* genes locate in 44 scaffolds, and their start position and end position are shown in Table 1. The candidate GRAS members were then uploaded to the CD-search website (https://www.ncbi.nlm.nih.gov/Structure/cdd/wrpsb.cgi) and their domain information were listed in Table 1, too. Besides, the gene structures of *JrGRAS* was presented in Fig. S1, and subcellular location information of the *Jr*GRAS proteins was presented in Table S1.

**Table 1:** *GRAS* gene family identified in *Juglans regia*.

Gene name	Gene symbol	Scaff	Scaff rename	Genome location	Strand	Related protein	Protein short name	GRAS domain position
*JrNSP2b*	*LOC108980261*	NW_017388857.1	Scaff2	NW_017388857.1: 818600–821800	+	XP_018806667.1	*Jr*NSP2b	119–499
*JrRGL1a*	*LOC108981380*	NW_017389446.1	Scaff13	NW_017389446.1: 52316–54604	−	XP_018808049.1	*Jr*RGL1a	155–526
*JrSCL32*	*LOC108982343*	NW_017443009.1	Scaff29	NW_017443009.1: 2200726–2203181	+	XP_018809230.1	*Jr*SCL32	51–453
*JrSCL28a*	*LOC108984412*	NW_017442835.1	Scaff27	NW_017442835.1: 99273–101699	+	XP_018811904.1	*Jr*SCL28a	303–669
*JrSCL1a-b*	*LOC108984751*	NW_017443020.1	Scaff30	NW_017443020.1: 188763–190597	−	XP_018812342.1	*Jr*SCL1a	202–571
						XP_018812343.1	*Jr*SCL1b	202–571
*JrSCL21a-d*	*LOC108985037*	NW_017439731.1	Scaff20	NW_017439731.1: 8737–10242	−	XP_018812730.1	*Jr*SCL21a	176–546
						XP_018812737.1	*Jr*SCL21b	176–546
						XP_018812742.1	*Jr*SCL21c	176–546
						XP_018812749.1	*Jr*SCL21d	176–546
*JrSCL23a*	*LOC108985505*	NW_017443560.1	Scaff36	NW_017443560.1: 1461510–1465205	+	XP_018813375.1	*Jr*SCL23a	73–427
*JrSCL14a*	*LOC108986374*	NW_017389857.1	Scaff15	NW_017389857.1: 63443–68155	+	XP_018814536.1	*Jr*SCL14a	369–740
*JrGAIc*	*LOC108986541*	NW_017440525.1	Scaff21	NW_017440525.1: 114274–115992	−	XP_018814727.1	*Jr*GAIc	245–603
*JrSCLa*	*LOC108987805*	NW_017443259.1	Scaff32	NW_017443259.1: 126697–128229	−	XP_018816363.1	*Jr*SCLa	1–310
*JrSCL3a*	*LOC108988066*	NW_017388959.1	Scaff7	NW_017388959.1: 519784–522043	−	XP_018816712.1	*Jr*SCL3a	40–422
*JrGAIb*	*LOC108988158*	NW_017389752.1	Scaff14	NW_017389752.1: 468309–470492	+	XP_018816848.1	*Jr*GAIb	238–599
*JrNSP2a*	*LOC108988310*	NW_017442823.1	Scaff26	NW_017442823.1: 505495–509211	+	XP_018817086.1	*Jr*NSP2a	117–501
*JrSHRa*	*LOC108988543*	NW_017443543.1	Scaff34	NW_017443543.1: 934500–937558	+	XP_018817374.1	*Jr*SHRa	105–484
*JrSCL3b*	*LOC108988679*	NW_017389863.1	Scaff16	NW_017389863.1: 241932–244037	+	XP_018817550.1	*Jr*SCL3b	44–457
*JrRGL1b*	*LOC108989561*	NW_017389020.1	Scaff10	NW_017389020.1: 713954–717041	−	XP_018818751.1	*Jr*RGL1b	138–508
*JrSCL27*	*LOC108990734*	NW_017389863.1	Scaff16	NW_017389863.1: 270998–273865	−	XP_018820345.1	*Jr*SCL27	379–740
*JrSCL18*	*LOC108992395*	NW_017389020.1	Scaff10	NW_017389020.1: 724451–727425	−	XP_018822504.1	*Jr*SCL18	47–445
*JrSHRb*	*LOC108992438*	NW_017389863.1	Scaff16	NW_017389863.1: 265516–268446	−	XP_018822539.1	*Jr*SHRb	60–433
*JrSCL14b*	*LOC108992934*	NW_017389020.1	Scaff10	NW_017389020.1: 761280–765445	+	XP_018823200.1	*Jr*SCL14b	325–701
*JrSCL13a*	*LOC108993395*	NW_017443546.1	Scaff35	NW_017443546.1: 1236377–1240941	+	XP_018823840.1	*Jr*SCL13a	176–546
*JrPAT1a-d*	*LOC108994062*	NW_017443598.1	Scaff41	NW_017443598.1: 206015–211096	−	XP_018824686.1	*Jr*PAT1a	168–538
						XP_018824687.1	*Jr*PAT1b	168–538
						XP_018824688.1	*Jr*PAT1c	168–538
						XP_018824689.1	*Jr*PAT1d	168–538
*JrSCL3c*	*LOC108994657*	NW_017443578.1	Scaff38	NW_017443578.1: 996644–998749	+	XP_018825500.1	*Jr*SCL3c	46–467
*JrSCL4a*	*LOC108995346*	NW_017388898.1	Scaff6	NW_017388898.1: 2412028–2413671	+	XP_018826448.1	*Jr*SCL4a	245–615
*JrSCL15*	*LOC108995362*	NW_017442540.1	Scaff24	NW_017442540.1: 34589–38721	−	XP_018826470.1	*Jr*SCL15	183–552
*JrSCLb-c*	*LOC108995898*	NW_017388887.1	Scaff4	NW_017388887.1: 1355328–1358341	−	XP_018827109.1	*Jr*SCLb	464–816
						XP_018827111.1	*Jr*SCLc	438–790
*JrSCLd*	*LOC108995938*	NW_017388969.1	Scaff8	NW_017388969.1: 382640–385001	+	XP_018827159.1	*Jr*SCLd	152–507
*JrSCL23b*	*LOC108996381*	NW_017388856.1	Scaff1	NW_017388856.1: 1066492–1069818	−	XP_018827796.1	*Jr*SCL23b	73–427
*JrSCL13b-d*	*LOC108996812*	NW_017388861.1	Scaff3	NW_017388861.1: 936197–939325	−	XP_018828372.1	*Jr*SCL13b	174–545
						XP_018828373.1	*Jr*SCL13c	174–545
						XP_018828374.1	*Jr*SCL13d	174–545
*JrSCL28b-c*	*LOC108997020*	NW_017442720.1	Scaff25	NW_017442720.1: 50539–52384	−	XP_018828642.1	*Jr*SCL28b	304–670
						XP_018828643.1	*Jr*SCL28c	304–643
*JrSCL4b*	*LOC108997571*	NW_017443591.1	Scaff40	NW_017443591.1: 871325–873488	+	XP_018829455.1	*Jr*SCL4b	252–623
*JrSCL3d-e*	*LOC108999242*	NW_017388893.1	Scaff5	NW_017388893.1: 2740337–2743452	+	XP_018831643.1	*Jr*SCL3d	46–468
						XP_018831644.1	*Jr*SCL3e	46–468
*JrRGL1c*	*LOC109001324*	NW_017442404.1	Scaff23	NW_017442404.1: 246803–250094	−	XP_018834108.1	*Jr*RGL1c	310–676
*JrSCL21e*	*LOC109001839*	NW_017443600.1	Scaff42	NW_017443600.1: 47744–51063	−	XP_018834825.1	*Jr*SCL21e	315–681
*JrSHRc*	*LOC109002462*	NW_017441391.1	Scaff22	NW_017441391.1: 33841–36019	−	XP_018835769.1	*Jr*SHRc	110–490
*JrSCL33a-b*	*LOC109002666*	NW_017443009.1	Scaff29	NW_017443009.1: 917876–919261	+	XP_018836065.1	*Jr*SCL33a	367–737
						XP_018836066.1	*Jr*SCL33b	367–737
*JrSCL34a*	*LOC109002667*	NW_017388893.1	Scaff5	NW_017388893.1: 730620–732711	+	XP_018836067.1	*Jr*SCL34a	383–753
*JrSCL9*	*LOC109002669*	NW_017443569.1	Scaff37	NW_017443569.1: 521973–524512	+	XP_018836069.1	*Jr*SCL9	386–757
*JrSCLe*	*LOC109004170*	NW_017443629.1	Scaff44	NW_017443629.1: 436277–438894	+	XP_018838179.1	*Jr*SCLe	449–801
*JrSLN1*	*LOC109006296*	NW_017437159.1	Scaff19	NW_017437159.1: 15109–18210	+	XP_018841073.1	*Jr*SLN1	151–518
*JrGAIa*	*LOC109007807*	NW_017443578.1	Scaff38	NW_017443578.1: 1317343–1319989	+	XP_018843202.1	*Jr*GAIa	226–585
*JrRGL1d*	*LOC109011259*	NW_017443604.1	Scaff43	NW_017443604.1: 879171–881593	+	XP_018847922.1	*Jr*RGL1d	156–525
*JrSCL22a*	*LOC109011601*	NW_017442999.1	Scaff28	NW_017442999.1: 59746–62789	+	XP_018848419.1	*Jr*SCL22a	438–793
*JrPAT1e-h*	*LOC109012627*	NW_017443590.1	Scaff39	NW_017443590.1: 1210837–1213906	+	XP_018849898.1	*Jr*PAT1e	176–546
						XP_018849899.1	*Jr*PAT1f	176–546
						XP_018849900.1	*Jr*PAT1g	176–546
						XP_018849901.1	*Jr*PAT1h	176–546
*JrSCL34b*	*LOC109013013*	NW_017443590.1	Scaff39	NW_017443590.1: 1237365–1238906	−	XP_018850468.1	*Jr*SCL34b	383–758
*JrSCL33c-d*	*LOC109013014*	NW_017389181.1	Scaff11	NW_017389181.1: 2256–5719	−	XP_018850470.1	*Jr*SCL33c	385–759
						XP_018850471.1	*Jr*SCL33d	385–759
*JrSCL14c*	*LOC109013019*	NW_017417453.1	Scaff18	NW_017417453.1: 8–1329	−	XP_018850477.1	*Jr*SCL14c	328–699
*JrCIGRa-b*	*LOC109014308*	NW_017443037.1	Scaff31	NW_017443037.1: 144211–146190	−	XP_018852274.1	*Jr*CIGRa	207–576
						XP_018852283.1	*Jr*CIGRb	207–576
*JrSCL22b*	*LOC109015811*	NW_017443532.1	Scaff33	NW_017443532.1: 1336635–1338234	+	XP_018853819.1	*Jr*SCL22b	386–747
*JrSLR1*	*LOC109015902*	NW_017389006.1	Scaff9	NW_017389006.1: 187337–190408	+	XP_018853896.1	*Jr*SLR1	151–449
*JrSCLf*	*LOC109017304*	NW_017389344.1	Scaff12	NW_017389344.1: 736656–738728	−	XP_018855146.1	*Jr*SCLf	1–309
*JrSCL3f*	*LOC109020246*	NW_017399977.1	Scaff17	NW_017399977.1: 832–2407	+	XP_018858211.1	*Jr*SCL3f	46–465

### Phylogenetic analysis of GRAS members

**Figure 1:**
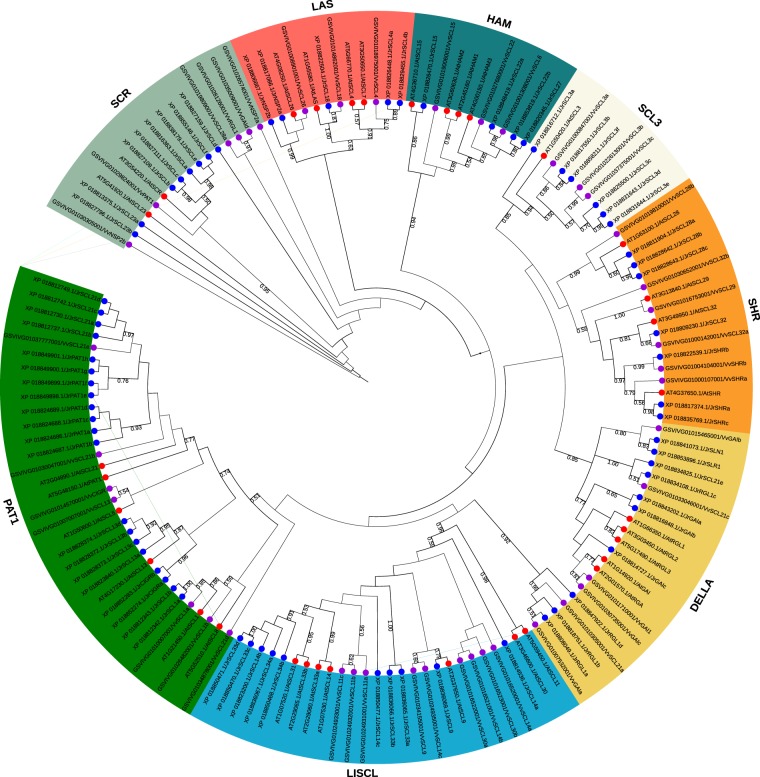
Phylogenetic tree of the domain sequence of GRAS proteins from Arabidopsis, walnut and grape using the Maximum Likelihood method. Genes in Arabidopsis, walnut and grape are labeled in red, blue and purple dots, respectively.

To study the phylogenetic relationships between GRAS family members in walnut, domain sequences of 70 walnut GRAS proteins, 33 Arabidopsis GRAS proteins and 43 grape GRAS proteins were used to construct an unrooted NJ phylogenetic tree in MEGA 6 with 1000 bootstrap replicates (Fig. 1). Based on the phylogenetic analysis and previous research^1^, all GRAS members were clustered into 8 subfamilies: PAT1, SCL3, DELLA, LAS, SCR, HAM, SHR, LISCL. The distribution of *Jr*GRAS proteins among different subfamilies was as following: PAT1(20), LISCL(14), DELLA (10), SCR(8), SCL3(6), HAM(4), LAS(4), and SHR(4).

### Definition the sequence features of *Jr*GRAS proteins

**Figure 2:**
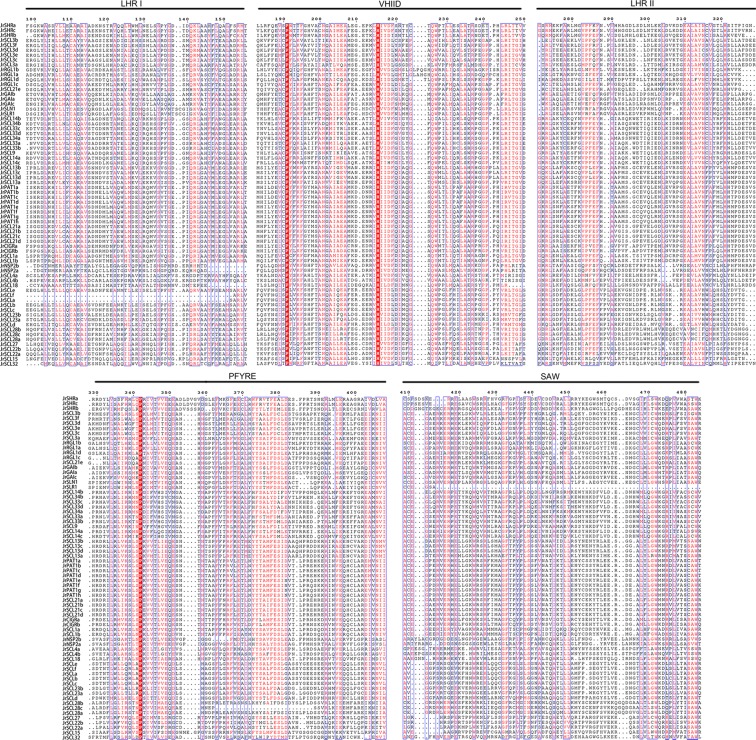
Alignment of the walnut GRAS protein sequences. The highly conserved regions of the *Jr*GRAS proteins were divided into five recognizable motifs.

The GRAS proteins in walnut share a highly conserved C-terminal, which is constituted by five distinct conserved motifs in the following order: LHR I (leucine heptad repeat I), VHIID, LHR II (leucine heptad repeat II), PFYRE and SAW, while the N-terminal region of the sequences seems to be variable (Fig. 2).

The presence of leucine heptad repeats in the GRAS proteins suggests that these proteins may function as multimers and a potentially complicated higher order of interaction^1^. The VHIID sequence consists of valine, histidine, isoleucine and aspartic acid, which is not absolutely conserved although it can be readily recognizable (position: 214–218, Fig. 2). Besides, we noticed the VHIID motif, the P residues (position: 191, Fig. 2) are absolutely conserved in the VHIID motif. The PFYRE motif consists of the P(position: 342)-F(position: 363)-Y(position: 374)-R(position: 366)-E(position: 369) (Fig. 2) residues, the P residues are absolutely conserved in PFYRE motif as well as in motif VHIID. The SAW motif is characterized by the residues S-A-W (position: 481–483, Fig. 2), the W(position: 472,483) residues are absolutely conserved in the other *Jr*GRAS protein sequences, except the *Jr*SLR1 which lack the SAW motif. And the absolute conservation of the residues in the VHIID and SAW motifs indicates that these residues could be necessary for the functions of the GRAS proteins.

### Conserved motifs analyses

**Figure 3:**
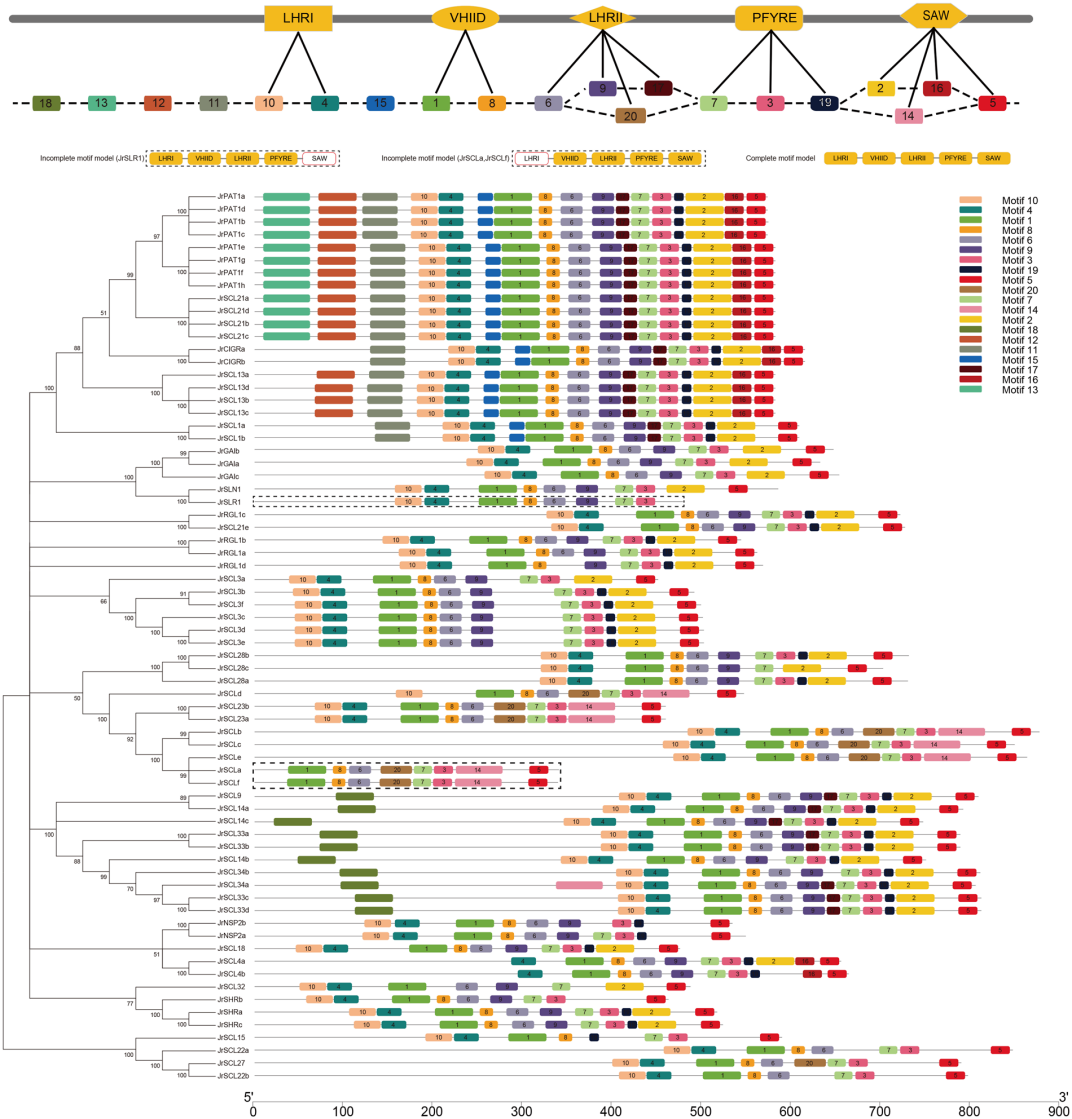
Phylogenetic relationship, motifs and gene structures of *GRAS* members in walnut.

All *Jr*GRAS proteins were subjected to MEME website (http://meme-suite.org/tools/meme)^49^ to identify conserved motifs (Fig. 3). Among the twenty Motifs, Motifs 10 and 4 consisted the LHR I domain, Motifs 1 and 8 consisted the VHIID domain, Motifs 6,9 and 17 or 6 and 20 consisted the LHR II domain, Motifs 7,3 and 19 consisted the PFYRE domain, and Motifs 2, 16 and 5 or 14 and 5 consisted the SAM domain (Fig. 3). Interesting, almost all *Jr*GRAS protein include the complete GRAS motif model, which consists of LHR I, VHIID, LHR II, PFYRE, and SAM domain, and the five domains distribute in the same order, except *Jr*SLR1, *Jr*SCLa and *Jr*SCLf.

### Synteny analysis and gene duplication of *JrGRAS* genes

#### Synteny analysis between different species

**Figure 4:**
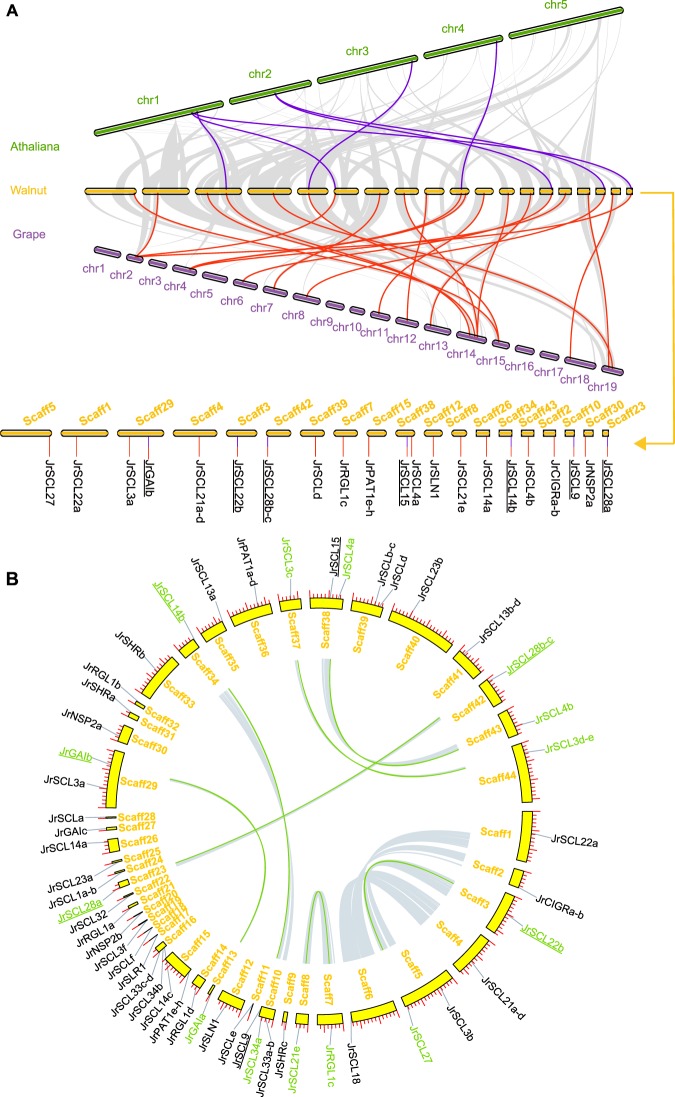
(**A**) Synteny analysis of *GRAS* genes between Arabidopsis, walnut and grape. The gray lines in the background indicate the collinear blocks within walnut and other plant genomes, while the blue and red lines highlight the syntenic GRAS gene pairs. (**B)** Synteny analysis of *JrGRAS* genes. Gray lines indicate all synteny blocks in the walnut genome, whereas the green lines suggest duplicated GRAS gene pairs. The gene name with an underline means this gene was identified as the synteny gene between different species.

To deduce the evolutionary relationship of *GRAS* genes between different species, syntenic analysis was performed for three plants (*A*. *thaliana*, *Vitis vinifera* and *Juglans regia*) (Fig. 4A). The result showed that there are many synteny blocks between Arabidopsis, grape and walnut. Among these blocks, seven walnut *GRAS* genes (*JrGAIb*/*JrSCL22b*/*JrSCL28b-c*/*JrSCL15*/*JrSCL14b*/*JrSCL9*/*JrSCL28a*) showed pairwise synteny with genes in Aradiposis genome, and twenty-one walnut *GRAS* genes (*JrSCL27*/*JrSCL22a*/*JrSCL3a*/*JrGAIb*/*JrSCL21a-d*/*JrSCL22b*/*JrSCL28b-c*/*JrSCLd*/*JrRGL1c*/*JrPAT1e-h*/*JrSCL15*/*JrSCL4a*/*JrSLN1*/*JrSCL21e*/*JrSCL14a*/*JrSCL14b*/*JrSCL4b*/*JrCIGRa-b*/*JrSCL9*/*JrNSP2a*/*JrSCL28a*) showed pairwise synteny with genes in grape genome. What is more, the seven walnut *GRAS* genes (*JrGAIb*/*JrSCL22b*/*JrSCL28b-c*/*JrSCL15*/*JrSCL14b*/*JrSCL9*/*JrSCL28a*) were identified to have orthologous genes within Aradiposis genome and within grape genome, simultaneously. These data indicated that the *GRAS* genes might have evolved from the common ancestor in different plants (The gene name with an underline means this gene was identified as the orthologous gene between different species).

#### Gene duplication in walnut genome

Gene duplication events were surveyed to explore the evolutionary patterns of the *GRAS* gene family in walnut genome (Fig. 4B). Physical locations of 52 *JrGRAS* genes in walnut were investigated by analysis of genomic distribution on scaffolds. Fifty-two *JrGRAS* genes were distributed unevenly across the 44 scaffolds in the walnut genome (Fig. 4B). Analysis of walnut *GRAS* family genes revealed seven paralogous gene pairs (*JrGAIa*&*JrGAIb/JrRGL1c*& *JrSCl21e/JrSCL14b*&*JrSCL34a/JrSCL22b*&*JrSCL27/JrSCL28a*&*JrSCL28b-c/JrSCL3c*&*JrSCL3d-e/JrSCL4a&JrSCL4b*) existed in walnut *GRAS* family genes. Among the 14 *GRAS* paralogous genes, 5 of them were orthologous genes identified between species, which indicated they were involved in the duplication event in walnut genome. (The gene name with an underline means this gene was identified as the orthologous gene between different species and the ‘&’ means connector between duplicated gene pairs).

### Expression profiles of *GRAS* members

We used the FPKM values of 52 *JrGRAS* genes to investigate the expression profiles of the *JrGRAS* family genes. Ten of the *JrGRAS* genes were excluded to draw the heatmap for their FPKM value were zero in both flower bud and leaf bud.

**Figure 5:**
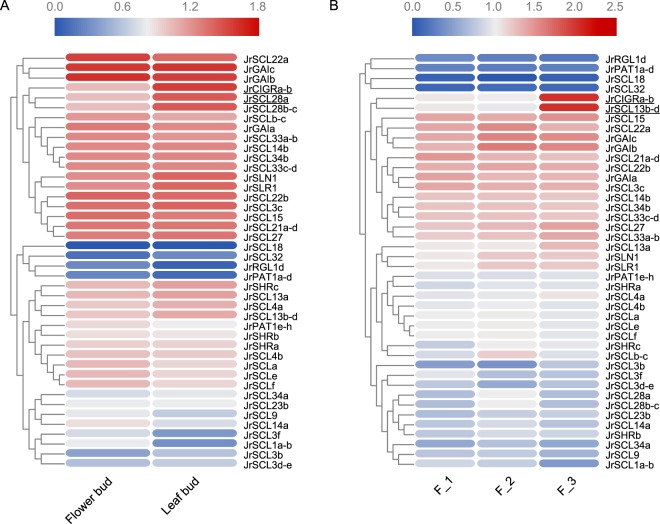
(**A)** Heatmap of the *JrGRAS* genes between flower buds and leaf buds. (**B)** Heatmap of *JrGRAS* genes expressed differently in three development periods of flower buds (F_1, F_2, and F_3).

First, expression levels of *JrGRAS* genes in female flower bud and in leaf bud were compared (Fig. 5A). Three *JrGRAS* genes (*JrSCL22a/JrGAIb/JrGAIc*) were highly expressed in both flower bud and leaf bud, and four *J*r*GRAS* genes (*JrSCL18/JrSCL32/JrRGL1d/JrPAT1a-d*) were lowly expressed in both flower bud and leaf bud. Besides, two *JrGRAS* genes (*JrCIGRa-b/JrSCL28a*) were differentially expressed between flower bud and leaf bud (p < 0.01).

Next, expression levels of the *JrGRAS* genes in female flower buds before, during, and after flower transition (F_1/F_2/F_3) were compared (Fig. 5B). Four *JrGRAS* genes (*JrSCL18*/*JrSCL32*/*JrRGL1d*/*JrPAT1a-d*) were lowly expressed in F_1, F_2 and F_3, and four *JrGRAS* genes (*JrSCL15*/*JrSCL22a*/*JrGAIb*/*JrGAIc*) were highly expressed in both flower bud and leaf bud. Besides, two *JrGRAS* genes (*JrCIGRa-b* and *JrSCL13b-d*) were differentially expressed between F_1, F_2 and F_3 (p < 0.01).

### GO enrichment

The GO enrichment analysis based on the 70 *Jr*GRAS proteins annotated in the GO database. In the biological process category, significantly enriched terms were associated with biological regulation, cellular process, metabolic process, and response to stimulus. In the cellular component category, cell, cell part, and organelle were significantly enriched. In the molecular function category, GO terms related to binding and nucleic acid binding transcription factor activity were highly represented. Besides, GO: 003674 (molecular function) was the most GO term enriched by the *JrGRAS* members (Fig. S2).

### Co-expression networks analysis of the *JrGRAS* family genes

**Figure 6:**
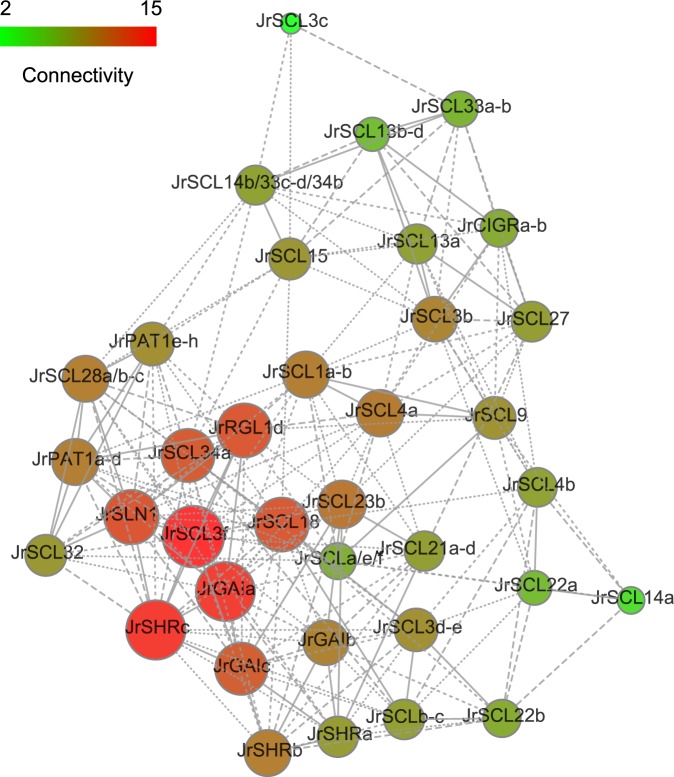
Co-expression networks of 58 *JrGRAS* genes. In the drawn weight network graph, the weight between genes will be divided into four parts, which are represented by point lines, short dotted lines, long dotted lines and real lines from small to large weights. The larger node and the redder color mean the greater connectivity of the gene in the network graph.

Weighted gene co-expression network analysis (WGCNA) is a biology method for interaction analysis and correlation networks resolving^50^. To search for the genes involved in flowering time regulation in walnut, *JrGRAS* family genes were used to construct a co-expression network with the method of WGCNA, the result was presented in Fig. 6. In the co-expression network, many of the key genes that participate in walnut flower bud transition were identified, such as *JrGAIa*, *JrSCL3f*, *JrSHRc*, *JrSCL34a*, *JrSLR1*, *JrRGL1d*, *JrSLN1*, *JrSCL18* and the hub genes with the highest edge numbers were *JrGAIa*, *JrSCL3f* and *JrSHRc*.

### Validation expression patterns of *JrGRASs* by qRT-PCR

**Figure 7:**
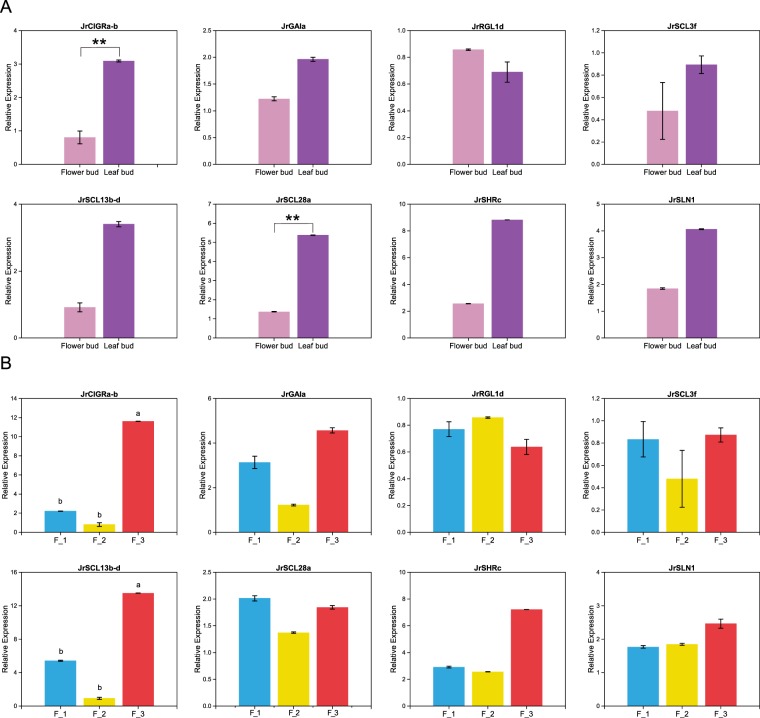
qRT-PCR analysis of *JrGRAS* genes in different tissues and different development period of flower buds.

The top five *JrGRAS* genes (*JrSCL3f*/*JrSHRc*/*JrGAIa*/*JrSLN1*/*JrRGL1d*) in the co-expression network and three DEGs (*JrCIGRa-b*, *JrSCL13b-d* and *JrSCL28a*) were used to conduct a qRT-PCR experiment (Fig. 7). The results were similar to those of our RNA-seq analysis and the *DEGs* were evidently differentially expressed among different tissues and development stages (P < 0.01). In leaf bud, *JrCIGRa-b* and *JrSCL28a* were all significantly up-regulated than that in flower bud (P < 0.01). As for flower bud transition periods, *JrCIGRa-b* and *JrSCL13b-d* were up-regulated in F_3 than that in F_1 and F_2 (P < 0.01). Among the *DEGs*, *JrCIGRa-b* differentially expressed in different tissues and different development period of flower buds, suggesting that this gene should work as the candidate gene for flower bud transition in walnut.

### Interaction network of *Jr*GRAS proteins

**Figure 8:**
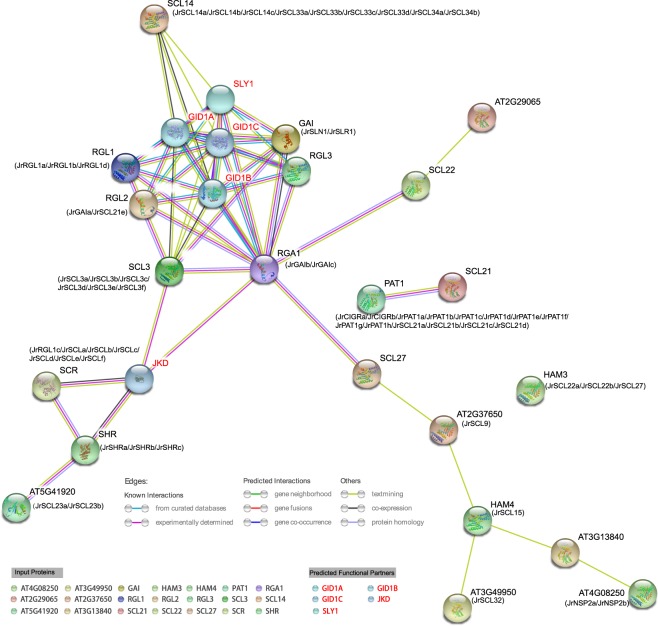
Predicted protein-protein interaction network of *Jr*GRAS proteins. The network nodes represent proteins, the 3D structure of the proteins is shown inside the nodes and the colors of the line indicate different data sources.

Because the interaction of walnut GRAS proteins is little known, we constructed the interaction network of the *Jr*GRAS proteins based on interaction relationship of the homologous GRAS proteins in Arabidopsis. The walnut GRAS proteins corresponded with the Arabidopsis GRAS proteins are listed below them (Fig. 8). The result showed that several *Jr*GRASs (such as *Jr*GAIb/*Jr*GAIc) were predicted to be core nodes in the network, which suggested that they might participate in diverse functions by interacting with other proteins.

## Discussion

In general, analysis of whole genome location and evolution rely on the available information of species genome assembled in Chromosomes-level. However, the walnut genome was assembled only in scaffold-level, and there is no access to the information of walnut Chromosomes until now. In this article, the 44 scaffolds which including the 52 *JrGRAS* genes were used to represent the walnut genome in the synteny and gene duplication analysis, and this may provide a new insight to the analysis of whole genome evolution for the species whose genome assembled in scaffold-level.

### Evolution of divergence and conservation

Divergence and conservation always come together with the process of species evolution. Phylogenetic analysis divided the *Jr*GRAS family into eight subgroups based on the evolutionary relationship, and each subgroup always function differently (Fig. 1). However, sequence alignment indicated that it was high conserved for the distribution of five motifs (LHR I, VHIID, LHR II, PFYRE, SAW motif) in *Jr*GRAS family members, and the order of these motifs within each protein is the same (Figs 2 and 3). Besides, in VHIID and SAW motifs, the absolute conserved residues suggested that these residues could be necessary for the activity of the GRAS proteins (Fig. 2).

### The duplication of *GRAS* genes between species and in walnut genome

Gene duplication between species indicated that Arabidopsis, walnut, and grape share the same seven ancestral *GRAS* genes. The number of orthologous genes of *GRAS* family genes in the three species showed a ratio of 7:21:21 (Arabidopsis: walnut: grape), which suggest a triplication event could occur in the *GRAS* family gene of walnut and grape. These caused us to further investigate the expansion of *GRAS* family gene in the walnut genome.

However, duplication analysis in walnut genome indicated that the triplicated speculation was invalid. Besides, duplicate genes face fates as follow: non-functionalization, neo-functionalization (evolving novel functions), or sub-functionalization (partition of gene functions)^51^. The seven orthologous *GRAS* genes (*JrGAIb*/*JrSCL22b*/*JrSCL28b-c*/*JrSCL15*/*JrSCL14b*/*JrSCL9*/*JrSCL28a*) occurred gene duplication event with function divergent in walnut genome, five of them duplicated with their pair genes (*JrGAIb*&*JrGAIa*/*JrSCL22b*&*JrSCL27*/*JrSCL28b-c*&*JrSCL28a*/*JrSCL14b*&*JrSCL34a*) still belong to the *GRAS* gene family. As for one of the five duplicated gene pairs mentioned above, (*JrSCL28b-c*&*JrSCL28a*), it seems that both of *JrSCL28b-c* and *JrSCL28a* come from the orthologous *GRAS* genes and this suggests that the duplication of them could be earlier than that in the other four duplicated gene pairs. What’s more, not all of the seven orthologous *GRAS* genes occurred gene duplication event, two of them (*JrSCL15*/*JrSCL9*) showed that they have no duplicated gene pairs in this research.

### Expression and function analysis of *JrGRAS genes*

*JrCIGRa-b* and *JrSCL28a* were identified to have a lower expression level in flower bud than that in leaf bud, which suggested these *JrGRAS* genes may negatively control the flower buds transition. And expression levels of *JrCIGRa-b* and *JrSCL13b-d* were detected up-regulated after flower buds transition (F_3) compared to that in (i) before the flower buds transition (F_1) and (ii) during the flower buds transition (F_2), which indicated that these *JrGRAS* genes may positively participate in the regulation of walnut flower organs development. Besides, three hub *JrGRAS* genes (*JrSCL3f*/*JrGAIa*/*JrSHRc*) were predicted by co-expression analysis, which suggested that they may involve in the regulation network of walnut flower buds transition, too.

Functional analysis of the *Jr*GRAS proteins seems to accord with the result of expression analysis. The GRAS domains are interacting with other domains identified by forming the heterodimer or homodimer structure. Up to now, two models of the GRAS domain interacting with other domains have been reported: (i) SHR-SCR heterodimeric structure; (ii) the homodimeric structure of the SCL7 GRAS domain^4^. And in this study, the SCR proteins (*Jr*SCLa/b/c/d/e/f) were predicted to interact with the SHR proteins (*Jr*SHRa/b/c) (Fig. 8), which consist with the SHR-SCR heterodimeric structure model. Besides, protein-protein interaction analysis showed that three hub *JrGRASs* (*JrSCL3f*/*JrGAIa*/*JrSHRc*) identified by expression analysis also have many interaction partners in the *Jr*GRAS protein-protein interaction network (Fig. 8), these results illustrate how *Jr*GRAS family proteins might form functional complexes, mediating the expression of flower bud transition genes in walnut.

Importantly, the *LAS* subfamily is involved and necessary in the growth regulation of the meristem formation^41,43,44^. A differentially expressed *JrGRAS* gene (*JrSCL28a*) in the *LAS* subfamily was found expressed both in leaf bud and flower bud, however, its expression level in leaf bud was significantly higher than that in flower bud (P < 0.01), the mechanism is still unclear. PAT1 is involved in phytochrome A signal transduction in Arabidopsis^35^. In this study, two *DEGs* (*JrCIGRa-b* and *JrSCL13b-d*, P < 0.01), identified (i) before, (ii) during and (iii) after flower bud transition (F_1, F_2 and F_3), were classified into the *PAT* subfamily, which indicated light signaling via the phytochrome A photoreceptor controls basic plant developmental processes, including flower bud development. Recently, a single walnut *GRAS* gene, *JrGRAS2* (*LOC108996381*, *JrSCL23b*, belongs to the *SCL* subfamily in this article), was reportedly involved in high-temperature stress tolerance^40^, which offer new insights to the functional diversity of walnut *GRAS* family members.

In summary, our work laid a foundation for future function investigation of the *GRAS* members in walnut and provides valuable information about the gene functions of *GRAS* family in the development of walnut flower bud transition.

## Methods

### Identification of *GRAS* family members in walnut

The latest protein sequences file (GCF_001411555.1_wgs.5d_protein.faa) of walnut genome was downloaded from the NCBI website (ftp://ftp.ncbi.nlm.nih.gov/genomes/all/GCF/001/411/555/GCF_001411555.1_wgs.5d/GCF_001411555.1_wgs.5d_protein.faa.gz). The hmm model of GRAS domain was constructed based on the PF03514 (PFAM website, http://pfam.xfam.org/family/pf03514) by the hmmbuild program HMMER 3.2^52^. Then, we used the hmm model mentioned above to search against the protein databases of walnut genomes with the hmmsearch program in HMMER 3.2^52^, the E-value cutoff was 1e−10. The candidate GRAS members were then uploaded to the CD-search website (https://www.ncbi.nlm.nih.gov/Structure/cdd/wrpsb.cgi) to further confirm if they include the proper GRAS domains (sequences included GRAS domain and length of domain sequences was more than 150aa). Gene structures of the *JrGRAS* genes were drawn by the Biosequence Structure Illustrator program of the TBtools software^53^. Subcellular location information of the *Jr*GRAS proteins was predicted by online software WoLF PSORT II (https://www.genscript.com/wolf-psort.html?src=leftbar).

### Multiple alignments and phylogenetic analyses

The domain sequences of the GRAS proteins in Arabidopsis, walnut and grape were downloaded from the Plant Transcription Factor Database (http://planttfdb.cbi.pku.edu.cn/) and aligned using Clustal X 2.1^54^. Then these sequences were used to conduct phylogenetic analyses using MEGA 6 software^55^ with 1000 bootstrap replicates. Motifs in the *Jr*GRAS family members were identified by MEME program (http://meme-suite.org/tools/meme)^49^ with a maximum of 20 motifs shown in the result.

### Synteny and gene duplication analysis

Analysis of gene duplication events using MCScanX toolkit^56^, paralogous genes in walnut genome were identified by the duplicate_gene_classifier program with the default parameters of the MCScanX toolkit, and orthologous genes between species were identified by the detect_collinear_tandem_arrays program with the default parameters of the MCScanX toolkit^56^. The genome sequences files and annotation files of Arabidopsis (RefSeq assembly accession: GCF_000001735.2, ftp://ftp.ncbi.nlm.nih.gov/genomes/all/GCF/000/001/735/GCF_000001735.4_TAIR10.1), walnut (RefSeq assembly accession: GCF_001411555.1) and grape (RefSeq assembly accession: GCF_000003745.3, ftp://ftp.ncbi.nlm.nih.gov/genomes/all/GCF/000/003/745/GCF_000003745.3_12X/) were downloaded from NCBI website (https://www.ncbi.nlm.nih.gov). The circle map of syntenic analysis maps in walnut genome was constructed by TBtools software^53^. Because of the walnut genome was assembled only in scaffold-level, the 44 scaffolds which including the 52 *JrGRAS* genes were used to represent the walnut genome in the synteny and gene duplication analysis.

### Expression analysis of *GRAS* members

Transcriptome sequencing and library construction were reported in our previous study^57^. Expression analysis of walnut *GRAS* members was evaluated using the walnut RNA-sequence data among different tissues (leaf bud and female flower bud), development stages (F_1, F_2, F_3). The FPKM values were normalized with the treatment of log10(FPKM), and the results were then used to generate heatmap using the HemI software^58^.

### RNA isolation and qRT-PCR analysis

The female flower buds were collected before, during, and after flower transition (F_1, F_2 and F_3), and leaf buds were collected during the floral transition period. The Leaf buds and female flower buds (F_1, F_2 and F_3) were collected and immediately frozen in liquid nitrogen. Total RNA was extracted with RNAout 1.0 (Tianenze, China) as described by the manufacturer and cDNA was reversed reverse-transcribed using the PrimeScript RT Reagent Kit (Takara, China). The real-time PCR analysis was performed using CFX Manager (Bio-Rad, USA) with SYBR Green mixture (Toyobo, Japan), and the walnut actin gene and walnut gadph gene were used for normalization, the amplification was applied using the cycling parameter as described previously^45^. The results were evaluated by the 2^−ΔCt^ method according to Livak and Schmittgen^59^.

### GO enrichment

The Blast2GO^60–63^ software was employed to perform the GO annotation. First, protein sequences of the *Jr*GRAS were used to perform the blastp search against the Swissport database with the E-Value of 1E-05, number of blast hits was 5. Then the result was conducted a GO mapping, and after that the GO annotation program was used to get the GO annotation of the *Jr*GRAS members. Finally, the GO enrichment analysis was conducted by the online GO enrichment program on the omicshare website (https://www.omicshare.com/tools/Home/Soft/gogsea).

### Interaction network of *Jr*GRAS proteins

The blastp program was used between the walnut GRAS proteins and the Arabidopsis GRAS proteins, each walnut GRAS protein matched a homologous Arabidopsis GRAS protein with the highest score (Table S7). Thirty-three Arabidopsis GRAS proteins which represent the 70 walnut GRAS proteins were uploaded to the String website (https://string-db.org/)^64^ to predict protein interactions. Except the 33 input proteins, five predicted functional partners of the input proteins were used to construct the network. The walnut GRAS proteins corresponded with the Arabidopsis GRAS proteins are listed below them. The online program ran with default parameters.

## Supplementary information

Dataset 1

## Data Availability

The data used to support the findings of this study are included in the article.
