# Supplementary material for: Genome-wide Identification, Classification, Expression and Duplication Analysis of GRAS Family Genes in Juglans regia L
Source: Sci Rep. 2019 Aug 12;9:11643. doi: 10.1038/s41598-019-48287-x (PMC6691012; doi:10.1038/s41598-019-48287-x)
Supplement: Supplementary file 1 — Dataset 1 [file 41598_2019_48287_MOESM1_ESM.doc]

Supplementary Information

**Genome-wide Identification, Classification, Expression and Duplication Analysis of *GRAS* Family Genes in *Juglans* *regia* L.**

**Shaowen Quan 1, 2, Jianxin Niu 1, 2*, Li Zhou 1, 2, Hang Xu1, 2, Li Ma1, 2, Yang Qin1, 2**

1 Department of Horticulture, College of Agriculture, Shihezi University, Shihezi 832003, Xinjiang, China.

2 Xinjiang Production and Construction Corps Key Laboratory of Special Fruits and Vegetables Cultivation Physiology and Germplasm Resources Utilization, Shihezi 832003, Xinjiang, China.

***Correspondence:** Jianxin Niu

**Email:** njx105@163.com

Address: Department of Horticulture, College of Agriculture, Shihezi University, Shihezi 832003, Xinjiang, China.

Supplementary Information

**Figure S1.** Gene structure of the *JrGRAS* genes.

**Table S1.** Subcellular location information of the *Jr*GRAS proteins.

**Table S2.** GRAS Subfamilies information of Arabidopsis and walnut.

**Table S3.** Synteny gene pair between species.

**Table S4.** Duplicated GRAS gene pairs in walnut genome.

**Table S5.** The FPKM Value of the walnut GRAS genes.

**Table S6.** Primers involved in this article.

**Table S7.** Walnut-Arabidopsis blastp result.

**
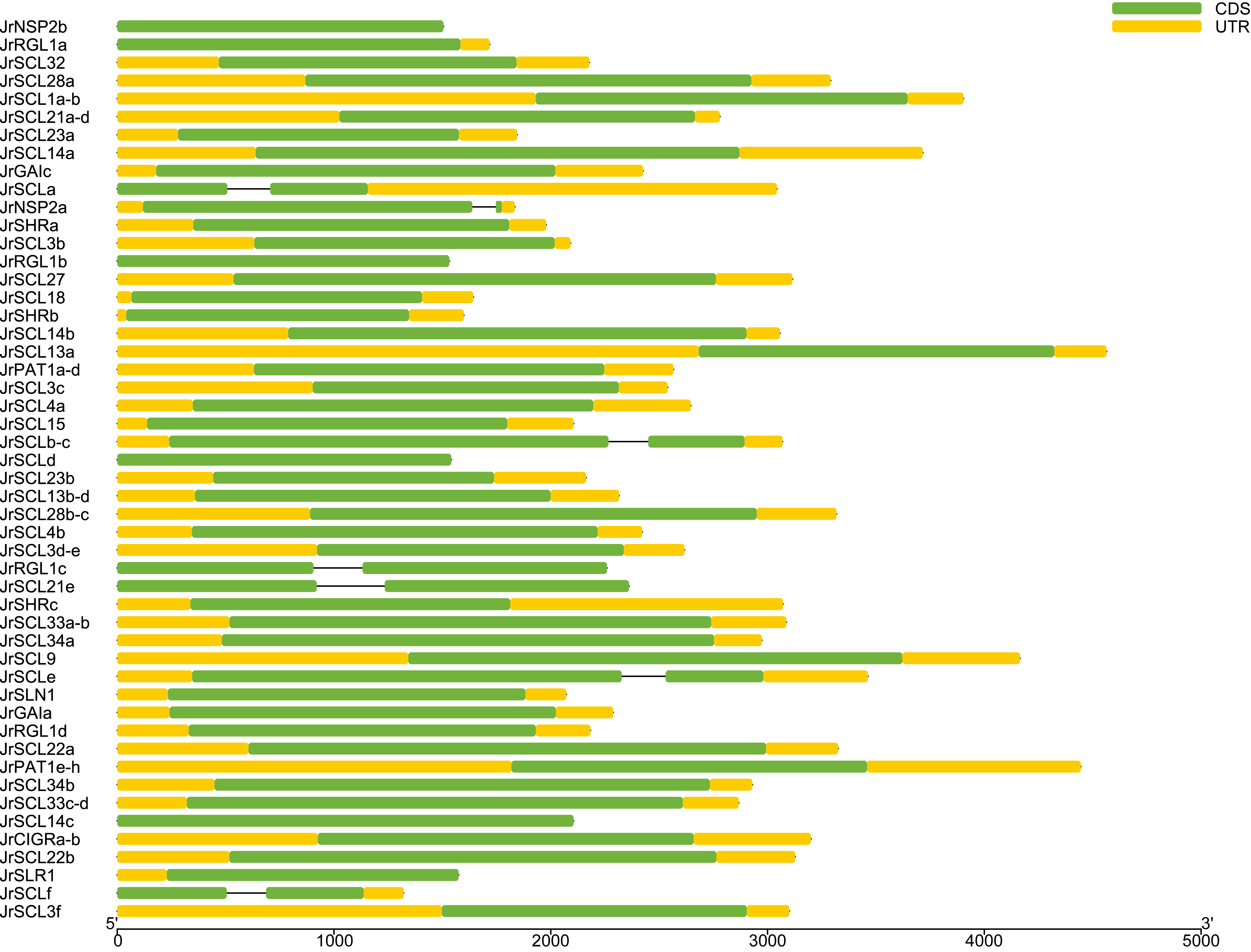
**

**Figure S1.** Gene structure of the *Jr*GRAS genes.

**
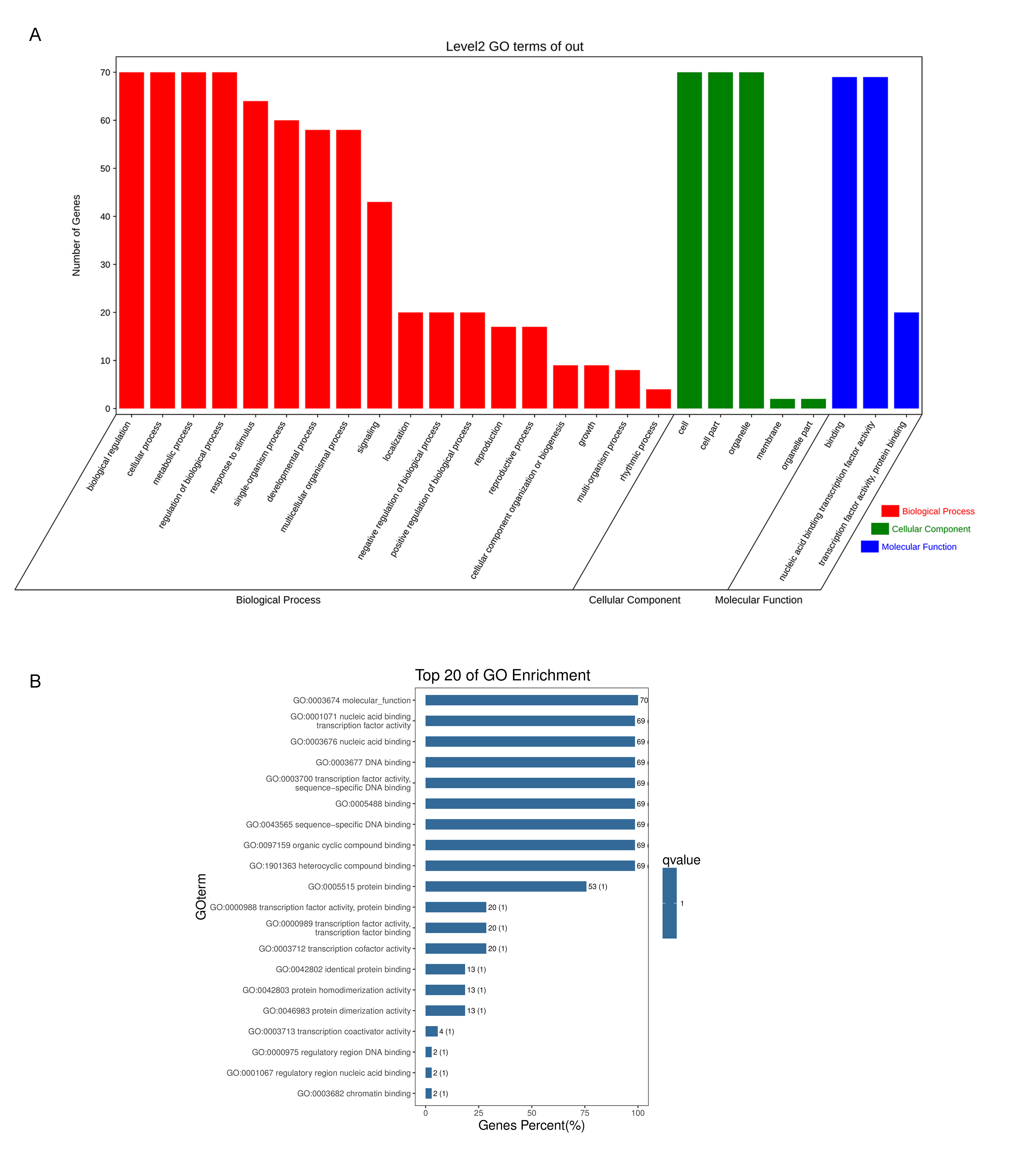
**

**Figure S2.** GO enrichment of the *Jr*GRAS genes. A. GO terms number of *Jr*GRAS members under biological process, cellular components and molecular function; B. Top20 enriched GO terms of the *Jr*GRAS members.

**Table S1.** **Subcellular location information of the *Jr*GRAS proteins.**

| Seq name | Gi number | Short name | Subcellular location (Plant) | Integral score | Multilocated |
| --- | --- | --- | --- | --- | --- |
| XP_018806667.1 | 1098801700 | *Jr*NSP2b | Nuclear | 7.3 | Nucleus_and_Endoplasmic_Reticulum |
| XP_018808049.1 | 1098804355 | *Jr*RGL1a | Extracellular(Secreted) | 2.4 | None |
| XP_018809230.1 | 1098806604 | *Jr*SCR32 | Nuclear | 8.7 | None |
| XP_018811904.1 | 1098811678 | *Jr*SCR28a | Nuclear | 8.4 | None |
| XP_018812342.1 | 1098812506 | *Jr*SCR1a | Cytoplasmic | 3.3 | Nucleus_and_Membrane |
| XP_018812343.1 | 1098812508 | *Jr*SCR1b | Cytoplasmic | 3.3 | Nucleus_and_Membrane |
| XP_018812730.1 | 1098728084 | *Jr*SCR21a | Cytoplasmic | 3.3 | Nucleus_and_Membrane |
| XP_018812737.1 | 1098728087 | *Jr*SCR21b | Cytoplasmic | 3.3 | Nucleus_and_Membrane |
| XP_018812742.1 | 1098728090 | *Jr*SCR21c | Cytoplasmic | 3.3 | Nucleus_and_Membrane |
| XP_018812749.1 | 1098728093 | *Jr*SCR21d | Cytoplasmic | 3.3 | Nucleus_and_Membrane |
| XP_018813375.1 | 1098814544 | *Jr*SCR23a | Nuclear | 8.8 | Cytoplasm_and_Nucleus |
| XP_018814536.1 | 1098816763 | *Jr*SCL14a | Nuclear | 8.8 | None |
| XP_018814727.1 | 1098817150 | *Jr*GAIc | Nuclear | 9 | None |
| XP_018816363.1 | 1098820347 | *Jr*SCRa | Nuclear | 8.8 | Cytoplasm_and_Nucleus |
| XP_018816712.1 | 1098821006 | *Jr*SCR3a | Cytoplasmic | 3.4 | None |
| XP_018816848.1 | 1098821263 | *Jr*GAIb | Nuclear | 9 | None |
| XP_018817086.1 | 1098821712 | *Jr*NSP2a | Nuclear | 7 | Nucleus_and_Endoplasmic_Reticulum |
| XP_018817374.1 | 1098822250 | *Jr*SHRa | Nuclear | 7.3 | Cytoplasm_and_Nucleus |
| XP_018817550.1 | 1098729431 | *Jr*SCR3b | Nuclear | 8.8 | Cytoplasm_and_Nucleus |
| XP_018818751.1 | 1098824839 | *Jr*RGL1b | Extracellular(Secreted) | 2.2 | None |
| XP_018820345.1 | 1098730357 | *Jr*SCR27 | Nuclear | 8.8 | None |
| XP_018822504.1 | 1098730740 | *Jr*SCR18 | Nuclear | 8.8 | None |
| XP_018822539.1 | 1098832100 | *Jr*SHRb | Nuclear | 7 | Cytoplasm_and_Nucleus |
| XP_018823200.1 | 1098833351 | *Jr*SCL14b | Nuclear | 8.8 | None |
| XP_018823840.1 | 1098834575 | *Jr*SCR13a | Cytoplasmic | 3.4 | Cytoplasm_and_Nucleus |
| XP_018824686.1 | 1098836188 | *Jr*PAT1a | Nuclear | 5.3 | Nucleus_and_Membrane |
| XP_018824687.1 | 1098836190 | *Jr*PAT1b | Nuclear | 5.3 | Nucleus_and_Membrane |
| XP_018824688.1 | 1098836192 | *Jr*PAT1c | Nuclear | 5.3 | Nucleus_and_Membrane |
| XP_018824689.1 | 1098836194 | *Jr*PAT1d | Nuclear | 5.3 | Nucleus_and_Membrane |
| XP_018825500.1 | 1098837728 | *Jr*SCR3c | Cytoplasmic | 3.3 | Cytoplasm_and_Nucleus |
| XP_018826448.1 | 1098839536 | *Jr*SCR4a | Nuclear | 8.5 | None |
| XP_018826470.1 | 1098839580 | *Jr*SCR15 | Nuclear | 8.3 | None |
| XP_018827109.1 | 1098840831 | *Jr*SCRb | Nuclear | 8.9 | Cytoplasm_and_Nucleus |
| XP_018827111.1 | 1098840833 | *Jr*SCRc | Nuclear | 8.8 | Cytoplasm_and_Nucleus |
| XP_018827159.1 | 1098840927 | *Jr*SCRd | Nuclear | 8.9 | Cytoplasm_and_Nucleus |
| XP_018827796.1 | 1098842136 | *Jr*SCR23b | Nuclear | 8.8 | Cytoplasm_and_Nucleus |
| XP_018828372.1 | 1098843257 | *Jr*SCR13b | Cytoplasmic | 3.4 | Cytoplasm_and_Nucleus |
| XP_018828373.1 | 1098843259 | *Jr*SCR13c | Cytoplasmic | 3.4 | Cytoplasm_and_Nucleus |
| XP_018828374.1 | 1098843261 | *Jr*SCR13d | Cytoplasmic | 3.4 | Cytoplasm_and_Nucleus |

**Table S1. Subcellular location information of the *Jr*GRAS proteins. (Continued)**

| XP_018828642.1 | 1098843791 | *Jr*SCR28b | Nuclear | 8.4 | None |
| --- | --- | --- | --- | --- | --- |
| XP_018828643.1 | 1098843793 | *Jr*SCR28c | Nuclear | 8.4 | None |
| XP_018829455.1 | 1098845305 | *Jr*SCR4b | Nuclear | 8.5 | None |
| XP_018831643.1 | 1098849567 | *Jr*SCR3d | Cytoplasmic | 3.2 | Cytoplasm_and_Nucleus |
| XP_018831644.1 | 1098849569 | *Jr*SCR3e | Cytoplasmic | 3.2 | Cytoplasm_and_Nucleus |
| XP_018834108.1 | 1098736772 | *Jr*RGL1c | Nuclear | 8.8 | Cytoplasm_and_Nucleus |
| XP_018834825.1 | 1098738360 | *Jr*SCR21e | Nuclear | 7.3 | None |
| XP_018835769.1 | 1098740467 | *Jr*SHRc | Nuclear | 7.3 | Cytoplasm_and_Nucleus |
| XP_018836065.1 | 1098741281 | *Jr*SCL33a | Nuclear | 8.8 | None |
| XP_018836066.1 | 1098741284 | *Jr*SCL33b | Nuclear | 8.8 | None |
| XP_018836067.1 | 1098741287 | *Jr*SCL34a | Nuclear | 8.9 | None |
| XP_018836069.1 | 1098741299 | *Jr*SCL9 | Nuclear | 8.9 | None |
| XP_018838179.1 | 1098747038 | *Jr*SCRe | Nuclear | 8.9 | Cytoplasm_and_Nucleus |
| XP_018841073.1 | 1098754564 | *Jr*SLN1 | Nuclear | 8.8 | None |
| XP_018843202.1 | 1098759769 | *Jr*GAIa | Nuclear | 9 | None |
| XP_018847922.1 | 1098774942 | *Jr*RGL1d | Nuclear | 7.3 | None |
| XP_018848419.1 | 1098722111 | *Jr*SCR22a | Nuclear | 8.5 | None |
| XP_018849898.1 | 1098778604 | *Jr*PAT1e | Cytoplasmic | 3.6 | Cytoplasm_and_Nucleus |
| XP_018849899.1 | 1098778606 | *Jr*PAT1f | Cytoplasmic | 3.6 | Cytoplasm_and_Nucleus |
| XP_018849900.1 | 1098778608 | *Jr*PAT1g | Cytoplasmic | 3.6 | Cytoplasm_and_Nucleus |
| XP_018849901.1 | 1098778610 | *Jr*PAT1h | Cytoplasmic | 3.6 | Cytoplasm_and_Nucleus |
| XP_018850468.1 | 1098779673 | *Jr*SCL34b | Nuclear | 8.7 | None |
| XP_018850470.1 | 1098779675 | *Jr*SCL33c | Nuclear | 8.8 | None |
| XP_018850471.1 | 1098779677 | *Jr*SCL33d | Nuclear | 8.8 | None |
| XP_018850477.1 | 1098779687 | *Jr*SCL14c | Nuclear | 8.8 | None |
| XP_018852274.1 | 1098723156 | *Jr*CIGRa | Nuclear | 5.5 | Nucleus_and_Membrane |
| XP_018852283.1 | 1098723159 | *Jr*CIGRb | Nuclear | 5.5 | Nucleus_and_Membrane |
| XP_018853819.1 | 1098723613 | *Jr*SCR22b | Nuclear | 8.8 | None |
| XP_018853896.1 | 1098780214 | *Jr*SLR1 | Nuclear | 8.8 | None |
| XP_018855146.1 | 1098786882 | *Jr*SCRf | Nuclear | 8.8 | Cytoplasm_and_Nucleus |
| XP_018858211.1 | 1098793581 | *Jr*SCR3f | Nuclear | 8.8 | Cytoplasm_and_Nucleus |

**Table S2.** GRAS Subfamilies information of Arabidopsis and walnut.

| **SPECIES** | **ID** | **NAME** | **DESCRIPTION** | **SUBFAMILY** | **COUNT** |
| --- | --- | --- | --- | --- | --- |
| **Arabidopsis** | AT5G59450.1 | AtSCL11 | GRAS family protein | LISCL | 7 |
|  | AT3G46600.1 | AtSCL30 | GRAS family protein |  |  |
|  | AT2G37650.1 | AtSCL9 | GRAS family protein |  |  |
|  | AT1G07530.1 | AtSCL14 | GRAS family protein |  |  |
|  | AT2G29060.1 | AtSCL33a | GRAS family protein |  |  |
|  | AT2G29065.1 | AtSCL33b | GRAS family protein |  |  |
|  | AT1G07520.1 | AtSCL31 | GRAS family protein |  |  |
|  | AT5G48150.1 | AtPAT1 | GRAS family protein | PAT1 | 6 |
|  | AT1G50600.1 | AtSCL5 | scarecrow-like 5 |  |  |
|  | AT2G04890.1 | AtSCL21 | SCARECROW-like 21 |  |  |
|  | AT1G21450.1 | AtSCL1 | SCARECROW-like 1 |  |  |
|  | AT4G17230.1 | AtSCL13 | SCARECROW-like 13 |  |  |
|  | AT5G52510.1 | AtSCL8 | GRAS family protein |  |  |
|  | AT3G03450.1 | AtRGL2 | RGA-like 2 | DELLA | 5 |
|  | AT1G14920.1 | AtGAI | GRAS family protein |  |  |
|  | AT2G01570.1 | AtRGA | GRAS family protein |  |  |
|  | AT1G66350.1 | AtRGL1 | RGA-like 1 |  |  |
|  | AT5G17490.1 | AtRGL3 | RGA-like protein 3 |  |  |
|  | AT4G36710.1 | AtSCL15 | GRAS family protein | HAM | 4 |
|  | AT3G60630.1 | AtHAM2 | GRAS family protein |  |  |
|  | AT4G00150.1 | AtHAM3 | GRAS family protein |  |  |
|  | AT2G45160.1 | AtHAM1 | GRAS family protein |  |  |
|  | AT5G66770.1 | AtSCL4 | GRAS family protein | SCL4/7 | 3 |
|  | AT4G08250.1 | AtSCL26 | GRAS family protein |  |  |
|  | AT3G50650.1 | AtSCL7 | GRAS family protein |  |  |
|  | AT4G37650.1 | AtSHR | GRAS family protein | SHR | 3 |
|  | AT3G49950.1 | AtSCL32 | GRAS family protein |  |  |
|  | AT3G13840.1 | AtSCL29 | GRAS family protein |  |  |
|  | AT1G55580.1 | AtLAS | GRAS family protein | LAS | 2 |
|  | AT1G63100.1 | AtSCL28 | SCARECROW-like 21 |  |  |
|  | AT3G54220.1 | AtSCR | GRAS family protein | SCR | 2 |
|  | AT5G41920.1 | AtSCL23 | GRAS family protein |  |  |
|  | AT1G50420.1 | AtSCL3 | scarecrow-like 3 | SCL3 | 1 |
| **Walnut** | XP_018812342.1 | JrSCL1a | scarecrow-like protein 1 | PAT1 | 20 |
|  | XP_018812343.1 | JrSCL1b | scarecrow-like protein 1 |  |  |
|  | XP_018812730.1 | JrSCL21a | scarecrow-like protein 21 |  |  |
|  | XP_018812737.1 | JrSCL21b | scarecrow-like protein 21 |  |  |
|  | XP_018812742.1 | JrSCL21c | scarecrow-like protein 21 |  |  |
|  | XP_018812749.1 | JrSCL21d | scarecrow-like protein 21 |  |  |
|  | XP_018823840.1 | JrSCL13a | scarecrow-like protein 13 |  |  |

**Table S2.** GRAS Subfamilies information of Arabidopsis and walnut. (continued)

| **Walnut** | XP_018824686.1 | JrPAT1a | scarecrow-like transcription factor PAT1 | PAT1 | 20 |
| --- | --- | --- | --- | --- | --- |
|  | XP_018824687.1 | JrPAT1b | scarecrow-like transcription factor PAT1 |  |  |
|  | XP_018824688.1 | JrPAT1c | scarecrow-like transcription factor PAT1 |  |  |
|  | XP_018824689.1 | JrPAT1d | scarecrow-like transcription factor PAT1 |  |  |
|  | XP_018828372.1 | JrSCL13b | scarecrow-like protein 13 |  |  |
|  | XP_018828373.1 | JrSCL13c | scarecrow-like protein 13 |  |  |
|  | XP_018828374.1 | JrSCL13d | scarecrow-like protein 13 |  |  |
|  | XP_018849898.1 | JrPAT1e | scarecrow-like transcription factor PAT1 |  |  |
|  | XP_018849899.1 | JrPAT1f | scarecrow-like transcription factor PAT1 |  |  |
|  | XP_018849900.1 | JrPAT1g | scarecrow-like transcription factor PAT1 |  |  |
|  | XP_018849901.1 | JrPAT1h | scarecrow-like transcription factor PAT1 |  |  |
|  | XP_018852274.1 | JrCIGRa | chitin-inducible gibberellin-responsive protein 1-like |  |  |
|  | XP_018852283.1 | JrCIGRb | chitin-inducible gibberellin-responsive protein 1-like |  |  |
|  | XP_018808049.1 | JrRGL1a | DELLA protein RGL1-like | DELLA | 10 |
|  | XP_018814727.1 | JrGAIc | DELLA protein GAIP-like |  |  |
|  | XP_018816848.1 | JrGAIb | DELLA protein GAI-like |  |  |
|  | XP_018818751.1 | JrRGL1b | DELLA protein RGL1-like |  |  |
|  | XP_018834108.1 | JrRGL1c | DELLA protein RGL1-like |  |  |
|  | XP_018834825.1 | JrSCL21e | scarecrow-like protein 21 |  |  |
|  | XP_018841073.1 | JrSLN1 | DELLA protein SLN1-like |  |  |
|  | XP_018843202.1 | JrGAIa | DELLA protein GAI-like |  |  |
|  | XP_018847922.1 | JrRGL1d | DELLA protein RGL1-like |  |  |
|  | XP_018853896.1 | JrSLR1 | DELLA protein SLR1-like |  |  |
|  | XP_018814536.1 | JrSCL14a | scarecrow-like protein 14 | LISCL | 10 |
|  | XP_018823200.1 | JrSCL14b | scarecrow-like protein 14 |  |  |
|  | XP_018836065.1 | JrSCL33a | scarecrow-like protein 33 |  |  |
|  | XP_018836066.1 | JrSCL33b | scarecrow-like protein 33 |  |  |
|  | XP_018836067.1 | JrSCL34a | scarecrow-like protein 34 |  |  |
|  | XP_018836069.1 | JrSCL9 | scarecrow-like protein 9 |  |  |
|  | XP_018850468.1 | JrSCL34b | scarecrow-like protein 34 |  |  |
|  | XP_018850470.1 | JrSCL33c | scarecrow-like protein 33 |  |  |
|  | XP_018850471.1 | JrSCL33d | scarecrow-like protein 33 |  |  |
|  | XP_018850477.1 | JrSCL14c | scarecrow-like protein 14 |  |  |
|  | XP_018813375.1 | JrSCL23a | scarecrow-like protein 23 | SCR | 8 |
|  | XP_018816363.1 | JrSCLa | protein SCARECROW-like |  |  |
|  | XP_018827109.1 | JrSCLb | protein SCARECROW-like |  |  |
|  | XP_018827111.1 | JrSCLc | protein SCARECROW-like |  |  |
|  | XP_018827159.1 | JrSCLd | protein SCARECROW-like |  |  |
|  | XP_018827796.1 | JrSCL23b | scarecrow-like protein 23 |  |  |

**Table S2.** GRAS Subfamilies information of Arabidopsis and walnut. (continued)

| **Walnut** | XP_018838179.1 | JrSCLe | protein SCARECROW-like | SCR | 8 |
| --- | --- | --- | --- | --- | --- |
|  | XP_018855146.1 | JrSCLf | protein SCARECROW-like |  |  |
|  | XP_018816712.1 | JrSCL3a | scarecrow-like protein 3 | SCL3 | 6 |
|  | XP_018817550.1 | JrSCL3b | scarecrow-like protein 3 |  |  |
|  | XP_018825500.1 | JrSCL3c | scarecrow-like protein 3 |  |  |
|  | XP_018831643.1 | JrSCL3d | scarecrow-like protein 3 |  |  |
|  | XP_018831644.1 | JrSCL3e | scarecrow-like protein 3 |  |  |
|  | XP_018858211.1 | JrSCL3f | scarecrow-like protein 3 |  |  |
|  | XP_018820345.1 | JrSCL27 | scarecrow-like protein 27 | HAM | 4 |
|  | XP_018826470.1 | JrSCL15 | scarecrow-like protein 15 |  |  |
|  | XP_018848419.1 | JrSCL22a | scarecrow-like protein 22 |  |  |
|  | XP_018853819.1 | JrSCL22b | scarecrow-like protein 22 |  |  |
|  | XP_018811904.1 | JrSCL28a | scarecrow-like protein 28 | LAS | 4 |
|  | XP_018822504.1 | JrSCL18 | scarecrow-like protein 18 |  |  |
|  | XP_018828642.1 | JrSCL28b | scarecrow-like protein 28 |  |  |
|  | XP_018828643.1 | JrSCL28c | scarecrow-like protein 28 |  |  |
|  | XP_018806667.1 | JrNSP2b | nodulation-signaling pathway 2 protein-like | SCL4/7 | 4 |
|  | XP_018817086.1 | JrNSP2a | nodulation-signaling pathway 2 protein |  |  |
|  | XP_018826448.1 | JrSCL4a | scarecrow-like protein 4 |  |  |
|  | XP_018829455.1 | JrSCL4b | scarecrow-like protein 4 |  |  |
|  | XP_018809230.1 | JrSCL32 | scarecrow-like protein 32 | SHR | 4 |
|  | XP_018817374.1 | JrSHRa | protein SHORT-ROOT-like |  |  |
|  | XP_018822539.1 | JrSHRb | protein SHORT-ROOT-like |  |  |
|  | XP_018835769.1 | JrSHRc | protein SHORT-ROOT-like |  |  |
| **Grape** | GSVIVG01016520001 | VvSCL14a | scarecrow-like protein 14 | LISCL | 9 |
|  | GSVIVG01016521001 | VvSCL14b | scarecrow-like protein 14 |  |  |
|  | GSVIVG01016522001 | VvSCL30a | scarecrow-like protein 30 isoform X1 |  |  |
|  | GSVIVG01016523001 | VvSCL30b | scarecrow-like protein 30 |  |  |
|  | GSVIVG01024931001 | VvSCL11a | SCARECROW-LIKE PROTEIN 11-RELATED |  |  |
|  | GSVIVG01024932001 | VvSCL11b | SCARECROW-LIKE PROTEIN 11-RELATED |  |  |
|  | GSVIVG01024933001 | VvSCL11c | SCARECROW-LIKE PROTEIN 11-RELATED |  |  |
|  | GSVIVG01024935001 | VvSCL14c | SCARECROW-LIKE PROTEIN 14-RELATED |  |  |
|  | GSVIVG01034155001 | VvSCL9 | SCARECROW-LIKE PROTEIN 9 |  |  |
|  | GSVIVG01007007001 | VvSCL13 | SCARECROW-LIKE PROTEIN 13 | PAT1 | 7 |
|  | GSVIVG01010007001 | VvSCL1a | SCARECROW-LIKE PROTEIN 1 |  |  |
|  | GSVIVG01014570001 | VvCIGR | chitin-inducible gibberellin-responsive protein 1-like |  |  |

**Table S2.** GRAS Subfamilies information of Arabidopsis and walnut. (continued)

| **Grape** | GSVIVG01026482001 | VvSCL1b | SCARECROW-LIKE PROTEIN 1 | PAT1 | 7 |
| --- | --- | --- | --- | --- | --- |
|  | GSVIVG01030047001 | VvSCL21b | SCARECROW-LIKE PROTEIN 21-RELATED |  |  |
|  | GSVIVG01034878001 | VvSCL21d | Scarecrow-like protein 21 |  |  |
|  | GSVIVG01037777001 | VvSCL21e | SCARECROW-LIKE PROTEIN 21-RELATED |  |  |
|  | GSVIVG01007532001 | VvGAIa | DELLA protein GAI | DELLA | 6 |
|  | GSVIVG01010305001 | VvSCL21a | scarecrow-like protein 21 |  |  |
|  | GSVIVG01011710001 | VvGAI1 | DELLA protein GAI1 |  |  |
|  | GSVIVG01015465001 | VvGAIb | Transcriptional regulator DELLA protein N terminal (DELLA) |  |  |
|  | GSVIVG01030735001 | VvGAIc | DELLA protein GAI-like |  |  |
|  | GSVIVG01033046001 | VvSCL21c | scarecrow-like protein 21 |  |  |
|  | GSVIVG01019809001 | VvSCL28a | SCARECROW-LIKE PROTEIN 28 | SCR | 6 |
|  | GSVIVG01026574001 | VvNSP2a | nodulation-signaling pathway 2 protein-like |  |  |
|  | GSVIVG01028126001 | VvRGL1 | DELLA protein RGL1-like |  |  |
|  | GSVIVG01029824001 | VvPAT1 | scarecrow-like transcription factor PAT1 |  |  |
|  | GSVIVG01030305001 | VvNSP2b | nodulation-signaling pathway 2 protein-like isoform X1 |  |  |
|  | GSVIVG01035009001 | VvGAId | DELLA protein GAIP-like |  |  |
|  | GSVIVG01004104001 | VvSHRb | Protein SHORT-ROOT | SHR | 4 |
|  | GSVIVG01016753001 | VvSCL29 | SCARECROW-LIKE PROTEIN 29 |  |  |
|  | GSVIVG01019810001 | VvSCL28b | SCARECROW-LIKE PROTEIN 28 |  |  |
|  | GSVIVG01030652001 | VvSCL32b | Scarecrow-like protein 32 |  |  |
|  | GSVIVG01001308001 | VvSCL6 | Scarecrow-like protein 6 | HAM | 3 |
|  | GSVIVG01019006001 | VvSCL15 | SCARECROW-LIKE PROTEIN 15 |  |  |
|  | GSVIVG01027680001 | VvSCL22 | SCARECROW-LIKE PROTEIN 22-RELATED |  |  |
|  | GSVIVG01008901001 | VvSCL26 | SCARECROW-LIKE PROTEIN 26 | LAS | 3 |
|  | GSVIVG01014862001 | VvSCL18 | SCARECROW-LIKE PROTEIN 18 |  |  |
|  | GSVIVG01018975001 | VvSCL4 | SCARECROW-LIKE PROTEIN 4-RELATED |  |  |
|  | GSVIVG01000847001 | VvSCL3a | Scarecrow-like protein 3 | SCL3 | 3 |
|  | GSVIVG01022613001 | VvSCL3b | Scarecrow-like protein 3 |  |  |
|  | GSVIVG01037375001 | VvSCL3c | Scarecrow-like protein 3 |  |  |
|  | GSVIVG01000107001 | VvSHRa | PROTEIN SHORT-ROOT | SHR | 2 |
|  | GSVIVG01000142001 | VvSCL32a | SCARECROW-LIKE PROTEIN 32 |  |  |

**Table S3.** Synteny gene pair between species.

**(gene name in bold means this gene have synteny gene pair in Arabidopsis and grape genome).**

| Arabidopsis | Walnut | Grape |
| --- | --- | --- |
| AT3G60630 | ***Jr*SCR22b** | LOC100267664 |
|  | *Jr*SCR27 | LOC100267664 |
| AT1G63100 | ***Jr*SCR28a** | LOC100247949 |
|  | *Jr*SCR22a | LOC100252271 |
|  | *Jr*RGL1c | LOC100263422 |
|  | *Jr*SCR21e | LOC100263422 |
| AT2G29060 | ***Jr*SCL14b** | LOC100262198 |
|  | *Jr*CIGRa-b | LOC100267541 |
|  | *Jr*SCR21a-d | LOC100250258 |
| AT2G29060 | ***Jr*SCL9** | LOC100252795 |
|  | *Jr*SLN1 | LOC100249084 |
|  | *Jr*PAT1e-h | LOC100261403 |
|  | *Jr*SCL14a | LOC100242700 |
| AT1G66350 | ***Jr*GAIb** | LOC100253268 |
|  | *Jr*SCR3a | LOC100263490 |
|  | *Jr*NSP2a | LOC100250491 |
| AT4G36710 | ***Jr*SCR15** | LOC100251313 |
|  | *Jr*SCR4a | LOC100246179 |
|  | *Jr*SCRd | LOC100263529 |
| AT1G63100 | ***Jr*SCR28b-c** | LOC100247949 |
|  | *Jr*SCR4b | LOC100246179 |

**Table S4.** Duplicated GRAS gene pairs in walnut genome.

**(gene name in bold means this gene have synteny gene pair in Arabidopsis and grape genome)**

| Walnut | Walnut |
| --- | --- |
| ***Jr*SCR22b** | *Jr*SCR27 |
| *Jr*RGL1c | *Jr*SCR21e |
| *Jr*SCL34a | ***Jr*SCL14b** |
| *Jr*GAIa | ***Jr*GAIb** |
| ***Jr*SCR28a** | ***Jr*SCR28b-c** |

**Table S5.** The FPKM Value of the walnut GRAS genes.

| **Gene** | **FPKM value** | | | | **q value** | | | | **UP_DOWN_REGULATION** | | | |
| --- | --- | --- | --- | --- | --- | --- | --- | --- | --- | --- | --- | --- |
|  | **F_1** | **F_2** | **F_3** | ***JR*L** | **F_1vsF_2** | **F_2vsF_3** | **F_1vsF_3** | **F_2vs*JR*L** | **F_1vsF_2** | **F_2vsF_3** | **F_1vsF_3** | **F_2vs*JR*L** |
| *Jr*SCR13b-d | 10.45 | 8.51 | 107.82 | 11.72 | 0.9999 | 9.18E-44 | 4.38E-41 | 0.99999 | FALSE | DOWN | DOWN | FALSE |
| *Jr*CIGRa-b | 10.42 | 9.71 | 106.91 | 36.47 | 0.9999 | 1.69E-47 | 1.76E-46 | 1.24E-07 | FALSE | DOWN | DOWN | DOWN |
| *Jr*GAIb | 20.36 | 41.62 | 35.8 | 35.46 | 0.011895 | 0.99975 | 0.17977 | 0.99999 | FALSE | FALSE | FALSE | FALSE |
| *Jr*GAIc | 23.43 | 37.6 | 34.15 | 39.34 | 0.24136 | 0.99975 | 0.82666 | 0.99999 | FALSE | FALSE | FALSE | FALSE |
| *Jr*SCR15 | 24.86 | 22.56 | 32 | 19.14 | 0.9999 | 0.99975 | 0.99997 | 0.99999 | FALSE | FALSE | FALSE | FALSE |
| *Jr*SCR27 | 15.92 | 18.9 | 25.27 | 20.52 | 0.9999 | 0.99975 | 0.62305 | 0.99999 | FALSE | FALSE | FALSE | FALSE |
| *Jr*SCL33a-b | 15 | 16.9 | 23.2 | 14.63 | 0.9999 | 0.99975 | 0.7492 | 0.99999 | FALSE | FALSE | FALSE | FALSE |
| *Jr*SCR22b | 21.76 | 24.35 | 22.24 | 22.76 | 0.9999 | 0.99975 | 0.99997 | 0.99999 | FALSE | FALSE | FALSE | FALSE |
| *Jr*SCR22a | 23.95 | 34.87 | 20.86 | 22.81 | 0.9999 | 0.55636 | 0.99997 | 0.99999 | FALSE | FALSE | FALSE | FALSE |
| *Jr*SCR3c | 23.92 | 21.58 | 20.8 | 24.5 | 0.9999 | 0.99975 | 0.99997 | 0.99999 | FALSE | FALSE | FALSE | FALSE |
| *Jr*GAIa | 25.29 | 19.45 | 19.52 | 16.28 | 0.9999 | 0.99975 | 0.99997 | 0.99999 | FALSE | FALSE | FALSE | FALSE |
| *Jr*SCR13a | 10.25 | 10.19 | 18.96 | 12.91 | 0.9999 | 0.63325 | 0.55064 | 0.99999 | FALSE | FALSE | FALSE | FALSE |
| *Jr*SCR21a-d | 29.62 | 20.12 | 16.91 | 20.05 | 0.9999 | 0.99975 | 0.36456 | 0.99999 | FALSE | FALSE | FALSE | FALSE |
| *Jr*SCL34b | 16.67 | 16.76 | 15.98 | 17.08 | 0.9999 | 0.99975 | 0.99997 | 0.99999 | FALSE | FALSE | FALSE | FALSE |
| *Jr*SCL33c-d | 16.67 | 16.76 | 15.98 | 17.08 | 0.9999 | 0.99975 | 0.99997 | 0.99999 | FALSE | FALSE | FALSE | FALSE |
| *Jr*SCL14b | 16.67 | 16.76 | 15.98 | 17.08 | 0.9999 | 0.99975 | 0.99997 | 0.99999 | FALSE | FALSE | FALSE | FALSE |
| *Jr*SLN1 | 10.23 | 15.78 | 15.38 | 23.79 | 0.9999 | 0.99975 | 0.99997 | 0.99999 | FALSE | FALSE | FALSE | FALSE |
| *Jr*SLR1 | 10.23 | 15.78 | 15.38 | 23.79 | 0.9999 | 0.99975 | 0.99997 | 0.99999 | FALSE | FALSE | FALSE | FALSE |
| *Jr*SCR4a | 7.92 | 8.42 | 11.05 | 11.02 | 0.9999 | 0.99975 | 0.99997 | 0.99999 | FALSE | FALSE | FALSE | FALSE |
| *Jr*SHRc | 5.29 | 9.81 | 8.34 | 13.03 | 0.9999 | 0.99975 | 0.99997 | 0.99999 | FALSE | FALSE | FALSE | FALSE |
| *Jr*SCRe | 9.22 | 9.89 | 8.1 | 7.32 | 0.9999 | 0.99975 | 0.99997 | 0.99999 | FALSE | FALSE | FALSE | FALSE |
| *Jr*SCRf | 9.22 | 9.89 | 8.1 | 7.32 | 0.9999 | 0.99975 | 0.99997 | 0.99999 | FALSE | FALSE | FALSE | FALSE |
| *Jr*SCRa | 9.22 | 9.89 | 8.1 | 7.32 | 0.9999 | 0.99975 | 0.99997 | 0.99999 | FALSE | FALSE | FALSE | FALSE |
| *Jr*SCR4b | 8.09 | 9.09 | 7.7 | 8.22 | 0.9999 | 0.99975 | 0.99997 | 0.99999 | FALSE | FALSE | FALSE | FALSE |
| *Jr*SHRa | 6.25 | 7.83 | 7.62 | 7.54 | 0.9999 | 0.99975 | 0.99997 | 0.99999 | FALSE | FALSE | FALSE | FALSE |
| *Jr*PAT1e-h | 7.38 | 7.12 | 7.58 | 5.54 | 0.9999 | 0.99975 | 0.99997 | 0.99999 | FALSE | FALSE | FALSE | FALSE |
| *Jr*SCRb-c | 6.63 | 14.33 | 7.31 | 11.96 | 0.23174 | 0.49795 | 0.99997 | 0.99999 | FALSE | FALSE | FALSE | FALSE |
| *Jr*SHRb | 4.8 | 6.63 | 5.94 | 6.15 | 0.9999 | 0.99975 | 0.99997 | 0.99999 | FALSE | FALSE | FALSE | FALSE |
| *Jr*SCR23b | 3.86 | 5.33 | 5.43 | 5.38 | 0.9999 | 0.99975 | 0.99997 | 0.99999 | FALSE | FALSE | FALSE | FALSE |
| *Jr*SCR3b | 2.03 | 1.55 | 4.66 | 2.8 | 0.9999 | 0.99975 | 0.99997 | 0.99999 | FALSE | FALSE | FALSE | FALSE |
| *Jr*SCR3f | 8.06 | 4.07 | 4.42 | 1.23 | 0.9999 | 0.99975 | 0.99997 | 0.99999 | FALSE | FALSE | FALSE | FALSE |
| *Jr*SCL14a | 4.68 | 6.63 | 4.36 | 4.19 | 0.9999 | 0.99975 | 0.99997 | 0.99999 | FALSE | FALSE | FALSE | FALSE |
| *Jr*SCR3d-e | 4.81 | 2.65 | 4.34 | 3.27 | 0.9999 | 0.99975 | 0.99997 | 0.99999 | FALSE | FALSE | FALSE | FALSE |
| *Jr*SCR28a | 3.91 | 9.42 | 4 | 27.16 | 0.30841 | 0.38967 | 0.99997 | 2.77E-05 | FALSE | FALSE | FALSE | DOWN |
| *Jr*SCR28b-c | 4.58 | 5.05 | 3.17 | 3.27 | 0.9999 | 0.99975 | 0.99997 | 0.99999 | FALSE | FALSE | FALSE | FALSE |
| *Jr*SCL9 | 2.53 | 4.17 | 2.51 | 4.95 | 0.9999 | 0.99975 | 0.99997 | 0.99999 | FALSE | FALSE | FALSE | FALSE |
| *Jr*SCL34a | 5.56 | 1.95 | 2.16 | 0.6 | 0.9999 | 0.99975 | 0.99997 | 0.99999 | FALSE | FALSE | FALSE | FALSE |
| *Jr*SCR1a-b | 2.29 | 1.96 | 2.08 | 2.47 | 0.9999 | 0.99975 | 0.99997 | 0.99999 | FALSE | FALSE | FALSE | FALSE |
| *Jr*PAT1a-d | 6.13 | 5.35 | 1.78 | 1.01 | 0.9999 | 0.99975 | 0.63508 | 0.44874 | FALSE | FALSE | FALSE | FALSE |
| *Jr*RGL1d | 0.92 | 0.77 | 0.82 | 0.26 | 0.9999 | 0.99975 | 0.99997 | 0.99999 | FALSE | FALSE | FALSE | FALSE |
| *Jr*SCR32 | 0.38 | 0.78 | 0.67 | 0.58 | 0.9999 | 0.99975 | 0.99997 | 0.99999 | FALSE | FALSE | FALSE | FALSE |

**Table S5.** The FPKM Value of the walnut GRAS genes. (Continued)

| **Gene** | **FPKM value** | | | | **q value** | | | | **UP_DOWN_REGULATION** | | | |
| --- | --- | --- | --- | --- | --- | --- | --- | --- | --- | --- | --- | --- |
|  | **F_1** | **F_2** | **F_3** | ***JR*L** | **F_1vsF_2** | **F_2vsF_3** | **F_1vsF_3** | **F_2vs*JR*L** | **F_1vsF_2** | **F_2vsF_3** | **F_1vsF_3** | **F_2vs*JR*L** |
| *Jr*SCR18 | 1.22 | 0.86 | 0.66 | 0.14 | 0.9999 | 0.99975 | 0.99997 | 0.99999 | FALSE | FALSE | FALSE | FALSE |
| *Jr*SCR23a | 0.87 | 0 | 0.65 | 0.51 | NA | NA | 0.99997 | NA | FALSE | FALSE | FALSE | FALSE |
| *Jr*NSP2a | 0.44 | 0 | 0.54 | 0 | NA | NA | 0.99997 | NA | FALSE | FALSE | FALSE | FALSE |
| *Jr*NSP2b | 0.26 | 0.35 | 0.3 | 0.86 | 0.9999 | 0.99975 | 0.99997 | 0.99999 | FALSE | FALSE | FALSE | FALSE |
| *Jr*RGL1a | 0.82 | 0.57 | 0.26 | 0.39 | 0.9999 | 0.99975 | 0.99997 | 0.99999 | FALSE | FALSE | FALSE | FALSE |
| *Jr*RGL1b | 0.17 | 0 | 0.15 | 0 | NA | NA | 0.99997 | NA | FALSE | FALSE | FALSE | FALSE |
| *Jr*RGL1c | 0 | 0.74 | 0.12 | 1.12 | NA | 0.99975 | NA | 0.99999 | FALSE | FALSE | FALSE | FALSE |
| *Jr*SCL14c | 0.11 | 0.32 | 0 | 0.18­ | 0.9999 | NA | NA | 0.99999 | FALSE | FALSE | FALSE | FALSE |
| *Jr*SCR21e | 0 | 0 | 0 | 0 | NA | NA | NA | NA | FALSE | FALSE | FALSE | FALSE |
| *Jr*SCR3a | 0 | 0 | 0 | 0 | NA | NA | NA | NA | FALSE | FALSE | FALSE | FALSE |
| *Jr*SCRd | 0 | 0 | 0 | 0 | NA | NA | NA | NA | FALSE | FALSE | FALSE | FALSE |

**Table S6. Primers involved in this article.**

| Sequence Definition | Sense Primer (5’-3’) | Anti-sense Primer (5’-3’) |
| --- | --- | --- |
| Cluster-14922.76273/*Jr*SCL28a | TGATGGATGAGATAGTGAGAG | GGTGGTGTAGCAGTATAGTAT |
| Cluster-14922.71810/*Jr*CIGRa-b | CAGTCTATTAGGTATGGATGGA | CGTGCCGATTGTAGTTCT |
| Cluster-14922.83097/*Jr*SCL13b-d | GCAAGAGTCCAACACCAA | ATAACAGCATTAGCCACAGA |
| Cluster-14922.66858/*Jr*GAIa | CTGCTGCTGCTTCTTCTT | GTGGTAACGGAGGAGGAT |
| Cluster-14922.17679/*Jr*SHRc | ACTCCACATCTCCGACTC | TCCATTCTGCTACCAATCTC |
| Cluster-14922.67763/*Jr*SCL3f | ATTCTGCCTCCACATCATC | GTCGCTCTATCGGTAATCTT |
| Cluster-14922.36201/*Jr*SLN1 | ACGGAACGGAATACACAAT | GCAAGAAGCGAATCACAAT |

**Table S7.** Walnut-Arabidopsis blastp result.

| Query gi | Query Seq | Short name | Blastp-hit | Short name |
| --- | --- | --- | --- | --- |
| gi1098801700 | XP_018806667.1 | *Jr*NSP2b | AT4G08250.1 | AtSCL26 |
| gi1098804355 | XP_018808049.1 | *Jr*RGL1a | AT1G66350.1 | AtRGL1 |
| gi1098806604 | XP_018809230.1 | *Jr*SCR32 | AT3G49950.1 | AtSCL32 |
| gi1098811678 | XP_018811904.1 | *Jr*SCR28a | AT1G63100.1 | AtSCL28 |
| gi1098812506 | XP_018812342.1 | *Jr*SCR1a | AT1G21450.1 | AtSCL1 |
| gi1098812508 | XP_018812343.1 | *Jr*SCR1b | AT1G21450.1 | AtSCL1 |
| gi1098728084 | XP_018812730.1 | *Jr*SCR21a | AT5G48150.1 | AtPAT1 |
| gi1098728087 | XP_018812737.1 | *Jr*SCR21b | AT5G48150.1 | AtPAT1 |
| gi1098728090 | XP_018812742.1 | *Jr*SCR21c | AT5G48150.1 | AtPAT1 |
| gi1098728093 | XP_018812749.1 | *Jr*SCR21d | AT5G48150.1 | AtPAT1 |
| gi1098814544 | XP_018813375.1 | *Jr*SCR23a | AT5G41920.1 | AtSCL23 |
| gi1098816763 | XP_018814536.1 | *Jr*SCL14a | AT1G07530.1 | AtSCL14 |
| gi1098817150 | XP_018814727.1 | *Jr*GAIc | AT2G01570.1 | AtRGA |
| gi1098820347 | XP_018816363.1 | *Jr*SCRa | AT3G54220.1 | AtSCR |
| gi1098821006 | XP_018816712.1 | *Jr*SCR3a | AT1G50420.1 | AtSCL3 |
| gi1098821263 | XP_018816848.1 | *Jr*GAIb | AT2G01570.1 | AtRGA |
| gi1098821712 | XP_018817086.1 | *Jr*NSP2a | AT4G08250.1 | AtSCL26 |
| gi1098822250 | XP_018817374.1 | *Jr*SHRa | AT4G37650.1 | AtSHR |
| gi1098729431 | XP_018817550.1 | *Jr*SCR3b | AT1G50420.1 | AtSCL3 |
| gi1098824839 | XP_018818751.1 | *Jr*RGL1b | AT1G66350.1 | AtRGL1 |
| gi1098730357 | XP_018820345.1 | *Jr*SCR27 | AT4G00150.1 | AtHAM3 |
| gi1098730740 | XP_018822504.1 | *Jr*SCR18 | AT1G55580.1 | AtLAS |
| gi1098832100 | XP_018822539.1 | *Jr*SHRb | AT4G37650.1 | AtSHR |
| gi1098833351 | XP_018823200.1 | *Jr*SCL14b | AT1G07530.1 | AtSCL14 |
| gi1098834575 | XP_018823840.1 | *Jr*SCR13a | AT4G17230.1 | AtSCL13 |
| gi1098836188 | XP_018824686.1 | *Jr*PAT1a | AT5G48150.1 | AtPAT1 |
| gi1098836190 | XP_018824687.1 | *Jr*PAT1b | AT5G48150.1 | AtPAT1 |
| gi1098836192 | XP_018824688.1 | *Jr*PAT1c | AT5G48150.1 | AtPAT1 |
| gi1098836194 | XP_018824689.1 | *Jr*PAT1d | AT5G48150.1 | AtPAT1 |
| gi1098837728 | XP_018825500.1 | *Jr*SCR3c | AT1G50420.1 | AtSCL3 |
| gi1098839536 | XP_018826448.1 | *Jr*SCR4a | AT5G66770.1 | AtSCL4 |
| gi1098839580 | XP_018826470.1 | *Jr*SCR15 | AT4G36710.1 | AtSCL15 |
| gi1098840831 | XP_018827109.1 | *Jr*SCRb | AT3G54220.1 | AtSCR |
| gi1098840833 | XP_018827111.1 | *Jr*SCRc | AT3G54220.1 | AtSCR |
| gi1098840927 | XP_018827159.1 | *Jr*SCRd | AT3G54220.1 | AtSCR |
| gi1098842136 | XP_018827796.1 | *Jr*SCR23b | AT5G41920.1 | AtSCL23 |
| gi1098843257 | XP_018828372.1 | *Jr*SCR13b | AT4G17230.1 | AtSCL13 |
| gi1098843259 | XP_018828373.1 | *Jr*SCR13c | AT4G17230.1 | AtSCL13 |
| gi1098843261 | XP_018828374.1 | *Jr*SCR13d | AT4G17230.1 | AtSCL13 |
| gi1098843791 | XP_018828642.1 | *Jr*SCR28b | AT1G63100.1 | AtSCL28 |

**Table S7.** Walnut-Arabidopsis blastp result. (Continued)

| gi1098843793 | XP_018828643.1 | *Jr*SCR28c | AT1G63100.1 | AtSCL28 |
| --- | --- | --- | --- | --- |
| gi1098845305 | XP_018829455.1 | *Jr*SCR4b | AT5G66770.1 | AtSCL4 |
| gi1098849567 | XP_018831643.1 | *Jr*SCR3d | AT1G50420.1 | AtSCL3 |
| gi1098849569 | XP_018831644.1 | *Jr*SCR3e | AT1G50420.1 | AtSCL3 |
| gi1098736772 | XP_018834108.1 | *Jr*RGL1c | AT3G54220.1 | AtSCR |
| gi1098738360 | XP_018834825.1 | *Jr*SCR21e | AT3G03450.1 | AtRGL2 |
| gi1098740467 | XP_018835769.1 | *Jr*SHRc | AT4G37650.1 | AtSHR |
| gi1098741281 | XP_018836065.1 | *Jr*SCL33a | AT1G07530.1 | AtSCL14 |
| gi1098741284 | XP_018836066.1 | *Jr*SCL33b | AT1G07530.1 | AtSCL14 |
| gi1098741287 | XP_018836067.1 | *Jr*SCL34a | AT1G07530.1 | AtSCL14 |
| gi1098741299 | XP_018836069.1 | *Jr*SCL9 | AT2G37650.1 | AtSCL9 |
| gi1098747038 | XP_018838179.1 | *Jr*SCRe | AT3G54220.1 | AtSCR |
| gi1098754564 | XP_018841073.1 | *Jr*SLN1 | AT1G14920.1 | AtGAI |
| gi1098759769 | XP_018843202.1 | *Jr*GAIa | AT3G03450.1 | AtRGL2 |
| gi1098774942 | XP_018847922.1 | *Jr*RGL1d | AT1G66350.1 | AtRGL1 |
| gi1098722111 | XP_018848419.1 | *Jr*SCR22a | AT4G00150.1 | AtHAM3 |
| gi1098778604 | XP_018849898.1 | *Jr*PAT1e | AT5G48150.1 | AtPAT1 |
| gi1098778606 | XP_018849899.1 | *Jr*PAT1f | AT5G48150.1 | AtPAT1 |
| gi1098778608 | XP_018849900.1 | *Jr*PAT1g | AT5G48150.1 | AtPAT1 |
| gi1098778610 | XP_018849901.1 | *Jr*PAT1h | AT5G48150.1 | AtPAT1 |
| gi1098779673 | XP_018850468.1 | *Jr*SCL34b | AT1G07530.1 | AtSCL14 |
| gi1098779675 | XP_018850470.1 | *Jr*SCL33c | AT1G07530.1 | AtSCL14 |
| gi1098779677 | XP_018850471.1 | *Jr*SCL33d | AT1G07530.1 | AtSCL14 |
| gi1098779687 | XP_018850477.1 | *Jr*SCL14c | AT1G07530.1 | AtSCL14 |
| gi1098723156 | XP_018852274.1 | *Jr*CIGRa | AT5G48150.1 | AtPAT1 |
| gi1098723159 | XP_018852283.1 | *Jr*CIGRb | AT5G48150.1 | AtPAT1 |
| gi1098723613 | XP_018853819.1 | *Jr*SCR22b | AT4G00150.1 | AtHAM3 |
| gi1098780214 | XP_018853896.1 | *Jr*SLR1 | AT1G14920.1 | AtGAI |
| gi1098786882 | XP_018855146.1 | *Jr*SCRf | AT3G54220.1 | AtSCR |
| gi1098793581 | XP_018858211.1 | *Jr*SCR3f | AT1G50420.1 | AtSCL3 |
